# Synthesis, Structural Characterization, Cytotoxicity, and Protein/DNA Binding Properties of Pyridoxylidene-Aminoguanidine-Metal (Fe, Co, Zn, Cu) Complexes

**DOI:** 10.3390/ijms241914745

**Published:** 2023-09-29

**Authors:** Violeta Jevtovic, Munirah Sulaiman Othman Alhar, Dejan Milenković, Zoran Marković, Jasmina Dimitrić Marković, Dušan Dimić

**Affiliations:** 1 Department of Chemistry, College of Science, University Ha’il, Ha’il 81451, Saudi Arabia; 2 Department of Science, Institute for Information Technologies, University of Kragujevac, Jovana Cvijića bb, 34000 Kragujevac, Serbia; 3 Faculty of Physical Chemistry, University of Belgrade, Studentski Trg 12–16, 11000 Belgrade, Serbia

**Keywords:** DFT, pyridoxylidene-aminoguanidine, BSA, HSA, cytotoxicity

## Abstract

Pyridoxylidene-aminoguanidine (PLAG) and its transition metal complexes are biologically active compounds with interesting properties. In this contribution, three new metal-PLAG complexes, Zn(PLAG)(SO_4_)(H_2_O)].∙H_2_O (Zn-PLAG), [Co(PLAG)_2_]SO_4_∙2H_2_O (Co-PLAG), and [Fe(PLAG)_2_]SO_4_∙2H_2_O) (Fe-PLAG), were synthetized and characterized by the X-ray crystallography. The intermolecular interactions governing the stability of crystal structure were compared to those of Cu(PLAG)(NCS)_2_ (Cu-PLAG) within Hirshfeld surface analysis. The structures were optimized at B3LYP/6-31+G(d,p)(H,C,N,O,S)/LanL2DZ (Fe,Co,Zn,Cu), and stability was assessed through Natural Bond Orbital Theory and Quantum Theory of Atoms in Molecules. Special emphasis was put on investigating the ligand’s stability and reactivity. The binding of these compounds to Bovine and Human serum albumin was investigated by spectrofluorometric titration. The importance of complex geometry and various ligands for protein binding was shown. These results were complemented by the molecular docking study to elucidate the most important interactions. The thermodynamic parameters of the binding process were determined. The binding to DNA, as one of the main pathways in the cell death cycle, was analyzed by molecular docking. The cytotoxicity was determined towards HCT116, A375, MCF-7, and A2780 cell lines. The most active compound was Cu-PLAG due to the presence of PLAG and two thiocyanate ligands.

## 1. Introduction

The field of organometallic chemistry is an important aid to the modern quest for anticancer agents, as some tumors are not responding to the current treatment. Upon the introduction of cisplatin in the 1960s, this area of research has developed tremendously with the inclusion of new metals and ligand systems [1,2]. Cisplatin is mainly connected to the low selectivity, high toxicity, and resistance that develops after use [3]. Therefore, many compounds containing Fe, Co, Zn, and Cu as bioactive metals have been synthesized, characterized, and later examined towards cancerous cell lines [4,5,6,7]. The biological activity of compounds is usually assessed towards DNA and transport proteins, bovine serum albumin (BSA), and human serum albumin (HSA) to elucidate possible anticancer mechanisms [8,9,10,11].

Schiff bases often manifest as potential anti-bacterial and antiviral drugs with significant biological activity, e.g., antitumor agents [12,13,14,15]. Aminoguanidine Schiff base, AG, is one of the most potent inhibitors of carbonyl stress and the occurrence of diabetes complications [16,17,18]. Recently, a systematic study of the thermodynamic stability of various Cu (II) complexes with aminoguanidine (AG) was performed, together with the study of its secondary antioxidant activity [19]. Vitamin B6 is unique in the group of B vitamins because it is included in the metabolism of all three primary macronutrients (proteins, carbohydrates, hydrates, and lipids) [20]. Interest in the complexation abilities of compounds from the vitamin B6 group, including pyridoxal (PL), increased after discovering that upon heating an aqueous solution of PL with certain amino acids [21] pyridoxamine can be obtained and that this non-enzymatic transamination is accelerated by the presence of Cu (II), Al(III), and Fe(III) ions, thanks to the formation of complexes with Schiff bases.

**Figure 1:**
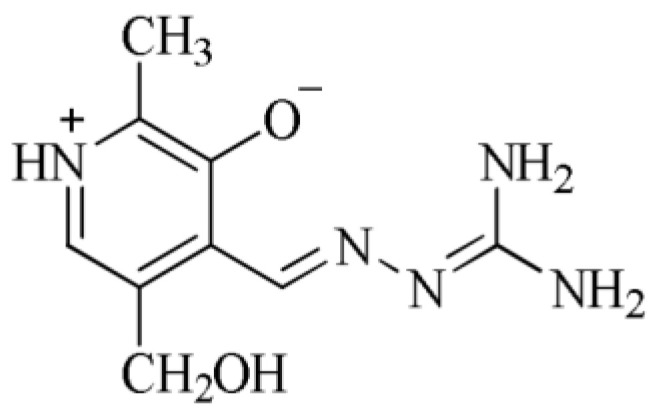
Structural formula PLAG.

**Figure 2:**
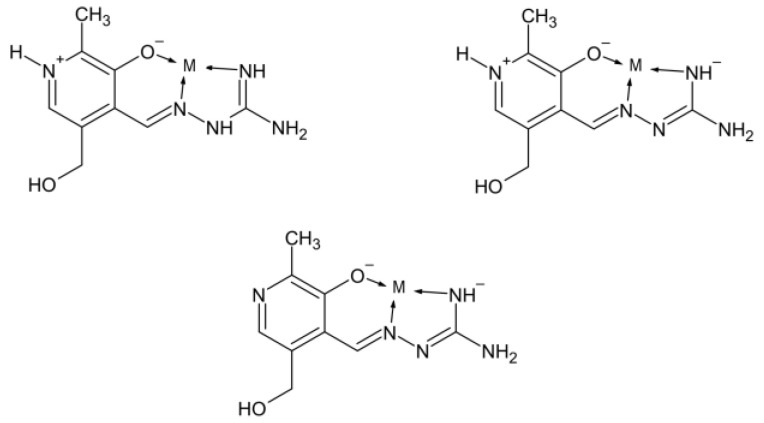
Different complexation modes of PLAG ligand.

In addition, research has shown that Schiff’s bases AG and pyridoxal can create together pyridoxylidene-aminoguanidine (PLAG) (Figure 1), which is superior to AG when treating diabetes complications is concerned because it does not only prevent lack of vitamin B6 but also allows better control of diabetes nephropathy [22,23,24]. Different complexation modes of PLAG, depending on protonation/deprotonation, are presented in Figure 2.

**Copper** ([Cu(PLAG)Cl_2_] [25], [Cu(PLAG)(MeOH)](NO_3_)_2_ [26], [Cu(PLAG)(NCS)_2_] [27], [Cu(PLAG–H)N_3_] [28], [Cu(PLAG-H)Cl]_2_(OAc)_2_·4H_2_O [29], [Cu(PLAG-H)Cl(NCS)]H_2_O [29], [Cu(PLAG)py(NO_3_)]NO [30], [{Cu(PLAG)py}](NO_3_)_4_ [30]), **Iron** ([Fe(PLAG)Cl_2_(H_2_O)]Cl [31], [Fe(PLAG)_2_](NO_3_)_3_ [31], **Cobalt** ([Co(PLAG-2H) (NH_3_)_3_]NO_3_MeOH [28], **Vanadium** (NH_4_[VO_2_(PLAG−2H)]·H_2_O [31], VO_2_(PLAG−H) [29], K[VO_2_(PLAG−2H)]·H_2_O [32]) and **Zinc** (PLAGH)_2_[ZnCl_4_] [33]) metal complexes with a ligand system (PLAG) have been synthesized so far.

It was shown that copper complexes of PLAG [29] bind to calf-thymus DNA with a binding constant comparable to ethidium bromide and cleavage pUC19 supercoiled plasmid DNA with activity of ∼99%. That is one of the rare studies [29] dealing with a broader overview and ability of the PLAG transition metal complexes. Otherwise, most papers describe only the synthesis and crystal structures of the PLAG complexes [25,26,27,28,30,31,32].

It turns out that more serious studies have been performed emphasizing the effect of PLAG itself and not its transition metal complexes. The effect of PLAG on preventing diabetic nephropathy in mice [24], on the glycation reaction of Aspartate Aminotransferase and Serum Albumin [34], the effect of PLAG to attenuate the abnormal aggregation of β-Tubulin and suppression of neurite outgrowth by Glyceraldehyde-derived toxic advanced glycation end-products [35] are some of these studies. 

The biological activity of PLAG transition-metal complexes, such as binding with transport proteins and DNA, has not received much attention.

Considering this issue, this contribution aimed to describe the synthesis of novel transition-metal (Fe, Co, Zn) complexes with PLAG, examine their crystallographic structure and intermolecular interactions through the Hirshfeld analysis, optimize structure by the Density Functional method (DFT) and assess the stabilization interactions through Natural Bond Orbital and Quantum Theory of Atoms in Molecules (QTAIM) approaches. The study also included the Cu-PLAG complex, obtained as described in the literature [27]. The binding activity towards BSA and HSA was analyzed by spectrofluorometric titration at three temperatures, and thermodynamic parameters of binding were calculated. The molecular docking study was performed to elucidate the interactions between PLAG complexes and amino acids of BSA/nucleobases of DNA. The cytotoxicity was determined towards colorectal carcinoma (HCT 116), melanoma (A375), breast cancer (MCF-7), and ovarian cancer (A2780). The special emphasis in the discussion was put on the effect of geometry and choice of metal ions on biological activity. 

## 2. Results

### 2.1. Crystalographic Structure Analysis

**Figure 3:**
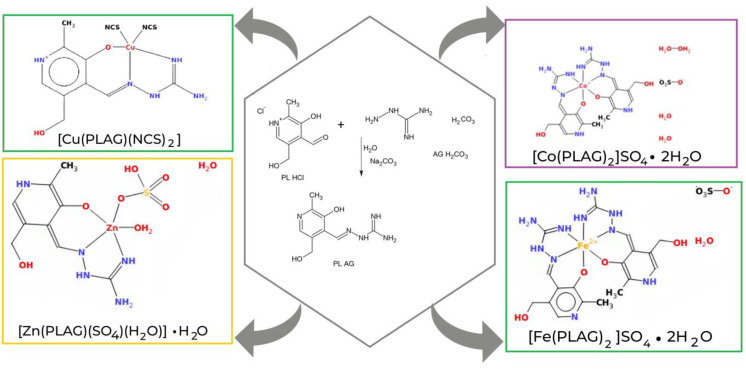
Structures of PLAG and metal-PLAG complexes. The color of squares represents colors of complexes.

The neutral form of the PLAG ligand was obtained according to the procedure described in the paper [16] by reaction between aqueous solutions of PL·HCl and AG·H_2_CO_3_ in a molar ratio of 1:1 and the presence of Na_2_CO_3_·10H_2_O (Figure 3). PLAG can coordinate as a tridentate ONN ligand via deprotonated oxygen atoms of phenolic groups and nitrogen atoms of azomethine and imino groups of AG fragment. In this way, two metallocycles are formed: one six-membered (pyridoxylidene—PL) and one five-membered (aminoguanidine—AG). The ligand PLAG appears as a zwitter ion (H2L), formed by the migration of hydrogen atoms from the phenolic OH-group to a pyridine nitrogen atom. Yellow microcrystals of PLAG ligand are soluble in MeOH and DMF. The ligand is stable in air and at elevated temperatures (the melting point is 152 °C). The molar conductivity of the ligand u MeOH corresponds to its non-electrolyte nature. 

For this paper, four PLAG transition metal complexes are analyzed, one of which was previously published (Cu(PLAG)(NCS)_2_ (**Cu-PLAG**) [16]). Newly synthesized complexes include [Zn(PLAG)(SO_4_)(H_2_O)]^.^H_2_O (**Zn-PLAG**), [Co(PLAG)_2_]SO_4_^.^2H_2_O (**Co-PLAG**), and [Fe(PLAG)_2_]SO_4_^.^2H_2_O) (**Fe-PLAG**) (Figure 3). These compounds contain PLAG ligands in the neutral form, which was also the criterion for selecting the Cu-PLAG complex. 

Cu-PLAG has a square pyramidal structure with an ONN PLAG ligand and coordinated SCN^−^ and NCS^−^ ions. Hydrogen on the pyridine nitrogen and azomethine nitrogen confirms the neutral form of PLAG. Three newly obtained complexes were synthesized using similar routes, including an aqueous solution of PLAG and zinc, cobalt, and iron sulfate salts. In these structures, metals have a double positive charge. The crystallization led to monocrystals further examined by the X-ray crystallographic analysis, as shown in the Methodology section. This is the first time that all three structures were obtained and described. The mono-ligand zinc complex has a square-pyramidal structure, with a tridentate ONN ligand PLAG, one sulfate ion, and a water molecule in coordination with zinc. There is and additional water molecule in a unit cell of Zn-PLAG. Cobalt and iron complexes are bis-ligand octahedral complexes containing only PLAG. Water molecules are present in unit cells of Co-PLAG and Fe-PLAG. Since sulfates were used as starting salts for the synthesis of all three complexes, it is interesting to monitor the behavior of the sulfate in the final structures. Although all three complexes were synthesized with a 1:1 salt: ligand molar ratio, the Co and Fe complexes are bis-ligands, i.e., the sulfate group is not coordinated with central metals. On the other side, SO_4_^2−^ forms a coordination bond with zinc. The reason may be the different sizes of the metal cations, which behave differently in contact with the bulky sulfate group. The intramolecular interactions are examined in detail in the following section. 

### 2.2. Hirshfeld Surface Analysis

The intramolecular interactions responsible for the stability of the investigated compounds are investigated by the Hirshfeld surface analysis. In the first place, those interactions characteristic of PLAG are discussed, and later, crystallographic structures of Zn-PLAG, as examples of newly obtained complexes, and Cu-PLAG are examined. The fingerprint plots for PLAG and two complexes are in Supplementary Materials as Figures S1–S3. 

**Figure 4:**
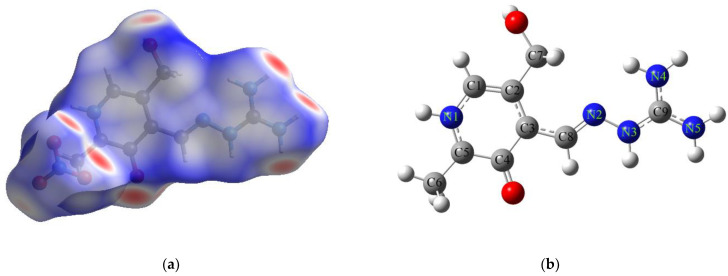
(**a**) Hirshfeld surface and (**b**) optimized structure (at B3LYP/6-311++G(d,p)) of PLAG (Hydrogen—white, carbon—gray, oxygen—red, nitrogen—blue).

The crystallographic structure of PLAG was obtained from the literature [27]. This structure was selected as the oxygen atom attached to the aromatic ring is deprotonated, and it resembles the structure of a ligand in the investigated complexes. PLAG consists of several functional groups that form various intermolecular interactions that are important for the stabilization of crystal packaging, as observed in Figure 4, depicting the Hirshfeld surface. The functional groups include the phenolic oxygen atom and hydroxymethyl group and the protonated nitrogen of the pyridine ring, hydrazine, and imine groups of the aliphatic chain. The crystallographic structure of PLAG also contains a nitro group as a counter ion. The highest contribution to overall interactions comes from O∙∙∙H (48.8%) due to the abundance of both elements in the crystal structure (Figure S1). These interactions are formed between amino and protonated pyridine moieties and counter ions, as well as between nitrogen-containing and hydroxymethyl groups. It can be assumed that these interactions are the strongest as two electronegative elements are included. The interactions between hydrogen atoms account for 27.1% due to the protonation of amino groups and pyridine nitrogen. Contacts between hydrogen and nitrogen atoms are much less common (4.7%) as all nitrogen atoms are protonated. The stabilization interactions denoted as C∙∙∙H (7.7%) are formed between hydrogen atoms and the π-electron cloud of the pyridine ring of two different PLAG molecules. The abundance of nitrogen atoms is also responsible for the high contribution of the following contacts: O∙∙∙N (6.0%) and C∙∙∙N (6.4%). The contributions from other interactions are negligible.

Upon complexation, the structure of PLAG is slightly distorted, and rotation around the C−C bond at the intersection between the pyridine ring and the aliphatic chain occurs. Zn-PLAG and Cu-PLAG were investigated by the Hirshfeld surface analysis as representative examples of crystallographic structures. The structure of Zn-PLAG contains one PLAG ligand, together with two water molecules and a sulfate group. Interestingly, one water molecule is coordinated to Zn while the second stabilizes the structure. On the other hand, the sulfate group is also coordinated with the central metal ion by forming the interaction between the lone pair of oxygen and empty Zn orbitals. As the central metal ion is surrounded by the ligands, there is only a small fraction of intermolecular interactions that include Zn, namely Zn∙∙∙H (0.1%) and Zn∙∙∙C (0.7%) (Figure S2). The highest contributions of interactions come from O∙∙∙H (44.0%) and H∙∙∙H (38.4%) since these atoms are positioned outwards the central metal ion and therefore interact with the surrounding units. Again, these interactions are mostly formed between hydrogen atoms attached to nitrogen and oxygen atoms of sulfate and hydroxymethyl groups. Hydrogen and carbon/nitrogen interactions contribute 4.2%/3.6% to the overall contacts. These values are lower than those obtained for PLAG as the different orientation of ligand in the crystal package is present. Interactions involving hydrogen also prove the importance of co-crystalized and coordinated solvents for the stability of crystals. This is also observed for N∙∙∙C, 3.5% compared to 6.4% in PLAG crystal. The square-pyramidal structure was also obtained in the case of Cu-PLAG instead of a water molecule, two SCN^−^ and NCS^−^ groups are present. The stabilization interaction between two Cu atoms accounts for 0.1%, while the interactions with surrounding nitrogen, denoted as Cu∙∙∙N, contribute 1.4% (Figure S3). Similar was previously shown for other Cu complexes [36,37]. Due to the existence of SCN^−^ and NCS^−^ groups in crystallographic structure, higher percentages of contacts between hydrogen and nitrogen/sulfur were calculated (S∙∙∙H (25.8%) and N∙∙∙H (15.6%)). The hydrogen–hydrogen interactions account for 18.3%, much lower than in the case of PLAG and Zn-PLAG. Stabilization contacts C∙∙∙H (15.9%) are formed between the pyridine ring and hydrogen atoms attached to electronegative atoms. The absence of oxygen atoms in ligands leads to a lower contribution of O∙∙∙H interactions (4.3%). The distribution of interactions between electronegative atoms is similar to Zn-PLAG, namely S∙∙∙N (3.7%), N∙∙∙O (3.5%), and S∙∙∙O (0.5%). The following sections investigate the intramolecular interactions responsible for the formation of complexes, the importance of different donor atoms, and the stability and reactivity of PLAG.

### 2.3. Theoretical Structural and Reactivity Analysis of PLAG

PLAG ligand has been extensively investigated for its complexation ability towards various metals [30], but also cytotoxicity and protein binding affinity [29,38,39]. Therefore, it is of outmost interest to analyze the structure and reactivity of this compound before moving towards its complexes with selected metals. The optimized structure of protonated PLAG at B3LYP/6-311++G(d,p) level of theory with enumeration scheme is shown in Figure 4b. To verify the applicability of the chosen level of theory, the bond lengths and angles of the optimized and crystallographic structure were compared by calculating the mean absolute error (MAE) between two sets of data [40]. These values are enlisted in Tables S1 and S2.

When bond lengths and angles were compared between theoretical and experimental structures of PLAG, the MAE values of 0.02 Å and 1.3° were obtained, respectively. These values prove that the selected level of theory was appropriate for the optimization of structure. The bond lengths are longer than the experimental ones within the experimental error due to the relaxation of the system during optimization; for example, the N1-C1 bond length was 1.35 in crystallographic and 1.37 Å in optimized structure. The theoretical analysis was performed for the isolated structure in a vacuum, which led to this discrepancy as no intermolecular interactions were present. When bond angles are concerned, the highest difference was observed for the angles at the intersection between the pyridine ring and aliphatic chain (123.48 vs. 126.57°) and within the hydrazine group (117.22 vs. 119.35°). Nevertheless, the results show that the optimized structure can be used for further analysis.

The intramolecular interactions are enlisted here and later compared with the optimized structures of complexes to explain any ligand’s structural change upon complexation. The stabilization energies were obtained through the Second-order perturbation theory and presented in Table S3. The structure of protonated PLAG is characterized by extended delocalization due to the presence of a pyridine ring, conjugated double bonds, and several atoms with lone pairs donated to the neighboring groups. Within the pyridine ring, the lone pair of nitrogen is already included in the electron delocalization due to the protonation of nitrogen. The interaction between N1-C5 and neighboring carbon-carbon bonds, denoted as π(N1-C5) → π*(C1-C2), has a stabilization energy of 61.4 kJ mol^−1^. The stabilization formed in the opposite direction, π(C1-C2) →π*(N1-C5), has a similar energy of 68.9 kJ mol^−1^. The phenolic oxygen stabilizes the structure of the ring by the interaction of lone pair with the surrounding C-C bonds through LP(O_phenolic_) → π*(C4-C5) (37.2 kJ mol^−1^). The same atom is also included in the interactions with the other bonds (π(O_phenolic_-C4) → π*(C5-N1) (43.4 kJ mol^−1^) and π(O_phenolic_-C4) → π*(C3-C4) (8.9 kJ mol^−1^)). The delocalization is also present in the hydroxymethyl group through the interaction of oxygen’s lone pair with the carbon–carbon bond (LP(O_hydroxymethyl_) → σ*(C2-C6), 37.2 kJ mol^−1^). The delocalization between the pyridine ring and aliphatic chain is facilitated through interaction σ(C3-C8) → σ*(N2-N3) (19.1 kJ mol^−1^). The abundance of nitrogen atoms in the aliphatic chain allows multiple interactions, especially in the hydrazine moiety (π(N2-C8) → π*(N4-C9) (33.0 kJ mol^−1^) and π(N3-C9) → π*(C8-N2) (45.0 kJ mol^−1^)). The strongest delocalization interactions are formed between two amino groups attached to the same carbon atom, namely LP(N4) → π*(C9-N3) (377.8 kJ mol^−1^) and LP(N5) → π*(C9-N3) (343.3 kJ mol^−1^). These stabilization interactions are important for the overall stability of the formed complexes, as ONN donor atoms are included.

**Figure 5:**
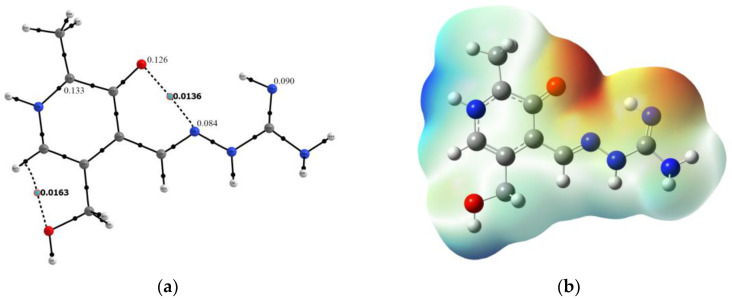
(**a**) Structure of PLAG with shown BCPs (dashed line) and CFFs and (**b**) MEP (at B3LYP/6-311++G(d,p), isovalue = 0.001 e/bohr^3^) of PLAG (Hydrogen—white, carbon—gray, oxygen—red, nitrogen—blue).

After the crystallographic data analysis of protonated PLAG structure, it is beneficial to access the reactivity of neutral PLAG present in the structure of obtained complexes. The proton removal was performed at the amino group, which resembles the experimental structure. Upon deprotonation, a rotation around the C3-C8 bond occurs, and phenolic oxygen is positioned on the same side as the amino group. The stability of this structure was accessed by the QTAIM investigation of the weak interactions formed within a structure. Based on the results shown in Figure 5a, two weak intramolecular interactions are formed. The first is a weak hydrogen bond between oxygen and the C-H group with an electron density of 0.0163 a.u and Laplacian of 0.0828 a.u. The second includes phenolic oxygen and hydrazine group (electron density 0.0136 a.u. and Laplacian 0.0521 a.u.). These open-shell interactions stabilize the structure of neutral PLAG. The reactivity of different positions within the molecule towards species with unpaired electrons was modeled by calculating the Condensed Fukui functions (CFF), as shown in the Methodology section [40,41]. The CFF values of the most reactive positions are presented in Figure 5a, while the complete list is given in Table S4. The highest values of the CFFs were obtained for the following atoms: C5 (0.133), O_phenolic_ (0.126), N2 (0.090), and N4 (0.084). Therefore, these positions are suggested as the most probable sites for interacting with radical species. It is important to outline that the positions of electronegative atoms (oxygen and nitrogen) coincide with those responsible for the complex compound formation, as explained in the following section. Atom C5 is in the vicinity of two electronegative atoms, and a change in electron density during protonation and deprotonation affects its NBO charge. The proposed methodology can be applied to predicting the reactive sites in newly obtained ligands [42]. A molecular electrostatic potential (MEP) map is a way to predict reactive sites for noncovalent interactions [43,44]. This methodology reflects the structural features of molecules, for example, lone pairs and strained bonds [45]. The electrostatic map of PLAG is shown in Figure 5b. The zone rich in electrons is positioned between phenolic oxygen, hydrazine group, and imino group. This area of a molecule is represented by red color. The abundance of electrons comes from the lone pairs of oxygen and nitrogen atoms in the area. This map explains the position of central metal ions and the complexation mode of PLAG. The differences in obtained complexes are examined in the following section.

### 2.4. Theoretical Analysis of Complexes

**Figure 6:**
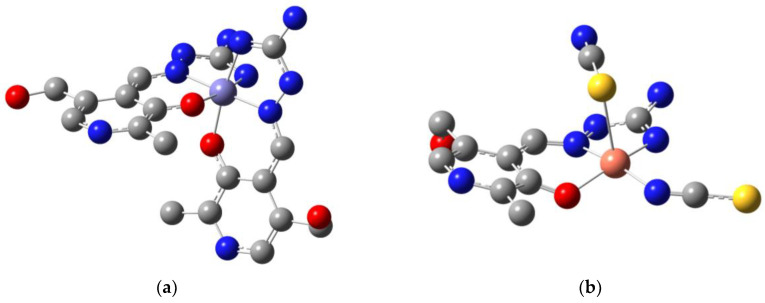
Optimized structures (at B3LYP/6-31+G(d,p)(H,C,N,O,S)/LanL2DZ(Fe,Cu)) of (**a**) Fe-PLAG and (**b**) Cu-PLAG. The hydrogen atoms are omitted for clarity. (Carbon—gray, oxygen—red, nitrogen—blue, sulfur—yellow, iron—purple, copper—coral).

The crystallographic structures of complexes were taken as starting geometries for the optimization at B3LYP/6-31+G(d,p)(H,C,N,O,S)/LanL2DZ(Zn,Fe,Co,Cu) level of theory. The experimental and theoretical bond lengths and angles are enlisted in Tables S5–S12 for selected structural parameters. The MAE values for these two data sets were calculated as previously explained. The optimized structures of Fe-PLAG and Cu-PLAG are shown in Figure 6 as representatives of complexes with octahedral and square pyramidal geometries.

Complexes containing two PLAG ligands are discussed first, as significant changes in the ligand structure are not expected. The MAE values for bond lengths and angles of the Fe-PLAG optimized and crystallographic structure are 0.04 Å and 7.5°. The bond lengths are well reproduced, as shown for the metal–ligand bonds. The Fe-O bond lengths are 1.94 and 1.96 Å, while in optimized, they are equal (1.98 Å). The average difference of Fe-N bond lengths is 0.14 Å, since the optimized bond lengths were longer in each case. These differences verify that the system was relaxed after the optimization. The lack of other compounds, as the optimizations were performed on a single compound in a vacuum, increased bond lengths. The importance of other compounds within the crystal unit, as presented in the Hirshfeld surface analysis, is verified through these calculations. Nevertheless, these values also show that the selected level of theory was appropriate for investigating the systems. Upon complexation, the bonds containing donor atoms were slightly changed or not changed at all, for example, C4-O 1.24 (PLAG) and 1.29 Å (Fe-PLAG). The C9-N4 bond length was 1.30 in both PLAG and Fe-PLAG. These values can be explained by the elongated delocalization within a structure that shortens the expected values of the bond length. Due to the complexation mode of PLAG, the structure is slightly twisted, but the average difference between PLAG bond lengths in free and bound ligands is 0.05 Å. The system’s relaxation is even more pronounced when bond angles are concerned, leading to the much higher MAE value of 7.5°. Careful inspection of the bond angles shows that the highest differences were obtained for the angles that contain two donor atoms and a central metal ion in Fe-PLAG. After the optimization, the pseudo-octahedral geometry was restored. For example, the O-Fe-O angle was 86.5° in the experimental and 90.3° in the optimized structure. Bond angles O-Fe-N show remarkable differences in some cases up to 20° (O2-Fe-N6), with the decrease in bond angle to 91.0°, a value expected for octahedral complexes. The spatial distribution of the donor atoms within the PLAG ligand allows the formation of these highly symmetric complexes. The angles of the ligand are not changed since this rigid structure is not easily distorted. The stabilization interactions are shown in Table S13. The electron donation occurs from the lone pair on carbonyl oxygen to the neighboring Fe-O interaction, LP(O_phenolic_) → σ*(Fe-O), with a stabilization energy of 82 kJ mol^−1^. The same type of electron density transfer was observed for the hydrazine nitrogen (LP(N_hydrazine_) → σ*(Fe-N), 18 kJ mol^−1^). The interactions between donor atoms and central metal ion additionally stabilize bonds within the PLAG structure, such as σ(Fe-N) → π*(N_imino_-C) (66 kJ mol^−1^).

In the case of Co-PLAG, the differences in experimental and theoretical bond lengths are even less pronounced (MAE values for bond lengths and angles are 0.03 Å and 2.17°). The reason for these values might be the shape of the crystal unit cell and the presence of co-crystalized solvent molecules. Upon optimization, the angles between donor atoms and central metal ions became closer to the idealized values, as in Table S8. The same types of stabilization interactions are present in this structure, namely LP(O_phenolic_) → σ*(Co-O) (64 kJ mol^−1^) and (LP(N_hydrazine_) → σ*(Co-N) (45 kJ mol^−1^). The lone pair of amino group nitrogen atom stabilizes other metal-donor atoms bonds such as LP(N_imino_) → σ*(Co-O) (12 kJ mol^−1^) and LP(N_imino_) → σ*(Co-N) (5 kJ mol^−1^). These results again prove that the ligand structure, as a tridentate ligand, is crucial for the stability of the obtained complex.

The comparison of bond lengths and angles of Cu-PLAG also verified that the selected level of theory was appropriate for describing the complex previously described in the literature. The MAE values for bond lengths and angles were 0.05 Å and 4.9°. Two thiocyanate ligands are coordinated through different atoms, namely sulfur and nitrogen. The experimental and theoretical donor atom–Cu bond lengths are 2.41 and 2.88 Å in the case of coordination through sulfur and 1.89 and 1.97 Å when coordination occurs through nitrogen. These values again prove that certain relaxation of the system occurs during the optimization process. The bond lengths between donor atoms and Cu are lower than in the case of Fe-PLAG, showing that the ligand is more tightly bound to this central metal ion. Upon optimization, the bond angles are much closer to the expected values for the square-pyramidal geometry; for example, the S-Cu-N angle was 121.0° and 96.7° in experimental and optimized structures, respectively. Moreover, the N_thiocyanate_-Cu-N_hydrazil_ angle is close to 180° in both structures. The thiocyanate ligands are almost linear, with the angle S-C-N being 171.6 (experimental) and 175.2° (theoretical). The structural features of PLAG are conserved after the complex formation. The lone pair of sulfur stabilizes the empty orbitals of Cu through LP(S) → LP*(Cu) (8 kJ mol^−1^). When interactions occur through the nitrogen atom of the thiocyanate ligand, the stabilization interaction is stronger (LP(N) → LP*(Cu), 167 kJ mol^−1^). It is important to notice that these stabilization interactions are comparable to those formed between PLAG and Cu, namely LP(O_phenolic_) → LP*(Cu) (103 kJ mol^−1^) and LP(O_phenolic_) → LP*(Cu) (179 kJ mol^−1^). The thiocyanate ligand is stabilized by the donation of electrons from the lone pair of sulfur to the neighboring C-N bond (LP(S) → π*(C-N), 156 kJ mol^−1^). The lone pair on nitrogen is also included in the stabilization of thiocyanate ion (LP(N) → π*(C-S), 59 kJ mol^−1^). When QTAIM analysis of the Cu-PLAG is concerned, the weakest interaction between donor atoms and Cu is formed in the case of Cu-S (electron density 0.0308 a.u. and Laplacian 0.0842 a.u.). The interaction between thiocyanate ions through nitrogen and Cu is stronger, with an electron density of 0.0740 a.u. The interactions between PLAG and Cu have the following electron densities: 0.0835 (O_phenolic_), 0.0689 (H_hydrazine_), and 0.1033 a.u. (H_imino_). These results show that QTAIM parameters can be used as a complementary approach to NBO analysis and that the electron density value reflects the strength of the interaction. These electron density values classify these interactions as much stronger than commonly observed weak interactions.

The optimized Zn-PLAG structure was also very similar to the crystallographic one, with MAE values for bond lengths and angles being 0.01 Å and 2.3°, respectively. The sulfate counter ion is part of the Zn-PLAG structure, and the bond length is 2.06 in the experimental structure. The stabilization interaction formed between sulfur of sulfate ion and Zn has a stabilization energy of 91 kJ mol^−1^ (Table S13). This value is comparable to that of the interaction including water molecule oxygen atom (LP(O_water_) → LP*(Zn), 80 kJ mol^−1^). The interaction between phenolic oxygen and Zn’s orbitals has a stabilization energy of 108 kJ mol^−1^, which is weaker than what was obtained for the interactions involving nitrogen atoms of PLAG (LP(N_hydrazine_) → LP*(Zn) (156 kJ mol^−1^) and LP(N_imino_) → LP*(Zn) (164 kJ mol^−1^). Due to the stability of the PLAG ligand, as explained previously, there are not significant changes in the stabilization interactions and geometrical parameters after the optimization. The interaction between the oxygen atom of the sulfate ion is characterized by the electron density of 0.0581 a.u. and Laplacian of 0.0310 a.u. The electron density value is higher than in the case of oxygen from the water molecule (electron density 0.0433 a.u.). The interactions between PLAG and Zn are weaker than in the case of Cu-PLAG, namely 0.0645 (O_phenolic_), 0.0586 (H_hydrazine_), and 0.0672 a.u. (H_imino_). The relative order of these interactions is the same in both compounds, proving that the interactions’ strength depends on the chosen metal.

### 2.5. Electronic Spectra and Stability of Complexes

The electronic spectrum of PLAG was recorded in water (Figure S5) between 250 and 1000 nm. The ligand spectrum consists of two doublet-like bands with maxima 310, 323, 346, and 358 nm. The intensity of the latter two is higher than for the first two maxima. This result is consistent with the literature, bearing in mind the possible shifts due to the solvent effect [31]. These maxima are assigned as π → π and n → π transitions within both pyridoxal and aminoguanidine moiety [30]. The addition of protons to the structure of PLAG leads to easier differentiation of these bands (~280 nm pyridoxal and ~340 nm aminoguanidine) [31]. Upon complexation, the bands belonging to the transitions within two parts of ligands are separated and shifted, depending on the metal ion and interaction strength. In the case of Co-PLAG, these bands are located at 293 and 364 nm, assigned to pyridoxal and aminoguanidine, respectively, as observed in the literature for similar compounds [31]. Due to the complexation, the second band is shifted for more than 20 nm. An even higher shift was obtained in the case of Cu-PLAG, with the bands located at 308 and 379 nm, consistent with the previous findings [30]. The spectra of Fe-PLAG and Zn-PLAG are characterized by the maxima at 357 and 416 nm due to the donor atom-central metal ion formation. It should be noted that the bands were widened as a result of charge transfer transitions that appeared as shoulders on higher wavelengths. The d-d bands were not observed in the spectra of complexes because of very low concentrations of complexes and the masking effect of other bands, as discussed in reference [28,31].

Prior to the protein binding affinity analysis, the stability of obtained compounds in aqueous and phosphate buffer saline solution was investigated, as decomposition of metal complexes can be expected under cell culture conditions [46,47]. The final concentration was set to 10^−4^ M, equal to the one used for the standard solution for spectrofluorimetric titrations. The stability was followed by the UV-VIS spectroscopy 30 min, 24, 48, 72 h, and 2 months after the preparation [48]. The UV-VIS spectra are presented as Figures S6 and S7. The spectra of complexes in water and phosphate buffer saline solutions are almost identical, although certain differences in absorbance value are present probably due to the present species in buffer solution. These spectra do not display significant changes during the time span selected for the stability studies. Therefore, these stock solutions were used for the experimental studies.

### 2.6. BSA Binding Affinity Analysis

The protein binding affinity of obtained compounds was analyzed by the spectrofluorometric titration of BSA and HSA solution by the solution containing obtained compounds. The mixture was irradiated by light with a wavelength of 295 (BSA) and 280 nm (HSA). Both proteins contain fluorescent amino acids within active pockets, and the binding process can be monitored through the change in intensity and position of the emitted light maxima. The fluorescent emission depends on the chemical environment of amino acids. The measurements for each complex were repeated at three temperatures to allow the determination of the thermodynamic parameters of binding. The following figure shows a representative example of the BSA titration by various concentrations of Zn-PLAG, and the obtained parameters for all compounds are summarized in Table 1.

**Figure 7:**
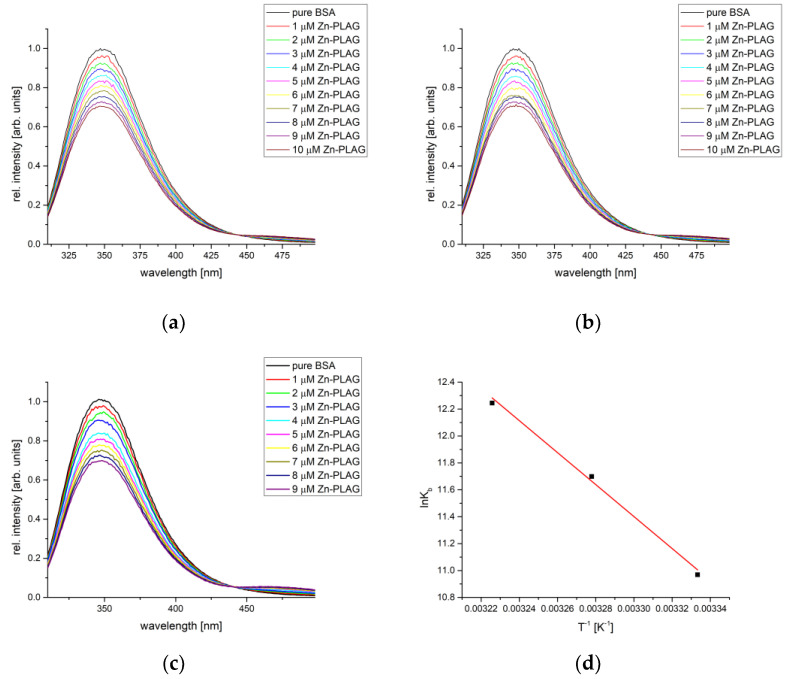
Fluorescence emission spectra of BSA for the titration with Zn-PLAG at (**a**) 27°, (**b**) 32°, (**c**) 37°, and (**d**) van’t Hoff plot for the binding process.

As seen in Figure 7, the fluorescence emission intensity of BSA decreased upon the addition of complexes in a concentration-dependent manner. The dependencies of fluorescence emission intensity vs. concentration according to Equation (2) were used to determine the binding constant and the number of binding positions. Table 1 also lists the correlation coefficients for this dependency, and their values were between 0.990 and 0.999, which proves that the Stern–Volmer double-log equation is applicable to the investigated systems. The number of binding places was between 0.96 and 1.13, showing that one complex compound bonded to one BSA molecule, forming M-PLAG-BSA (M = Zn,Co,Fe,Cu) species. In the case of newly obtained compounds, the binding constants were of the order 10^4^–10^5^ M^−1^, although their values depended on temperature.

When one molecule of PLAG is present, in the case of Zn-PLAG, the change in entropy and enthalpy are 98.78 kJ mol^−1^ and 420.77 J mol^−1^ K^−1^, respectively. The positive change in these two parameters indicates an entropy-driven process mostly reflecting a loss of rotational and translational degrees of freedom within the protein structure. It is important to mention that this complex can form various interactions as the sulfate group is part of the inner sphere, along with the PLAG molecule. As explained in the following paragraphs, these species can form hydrogen bonds with the surrounding amino acids. The spontaneity of the binding process increases with temperature (−27.45 to −31.66 kJ mol^−1^). A similar was observed for the Zn–sulfonamide complexes [49].

Complexes containing two PLAG ligands show similar behavior. The binding constants are of the same order of magnitude for both Co-PLAG and Fe-PLAG. The change in thermodynamic parameters in the case of Co-PLAG is much less pronounced than for Fe-PLAG (for example, changes in entropy are 16.015 and 154.38 kJ mol^−1^, respectively). As both of these processes are entropy-driven, it can be assumed that interactions with surrounding amino acids loosen the interactions with central metal ions, thus increasing the system’s overall entropy. As previously shown, the interactions between donor atoms and Fe are much stronger than those with Co. Both PLAG molecules in these complexes form interactions with amino acids through the same groups as Zn-PLAG, although the counter ion is not present. The changes in Gibbs free energy of Fe-PLAG binding are −25.82, −28.82, and −31.82 kJ mol^−1^ for the examined temperature range. These values are comparable to the Fe-pyridoxal-thiosemicarbazone complex containing this ligand molecule and two chlorido and one aqua ligand [37]. The static quenching mechanism is responsible for the decrease in the fluorescence intensity, as previously described for Fe complexes with Schiff bases [50]. The spontaneity of binding is the lowest in the case of Co-PLAG, which shows the importance of metal ions, besides the present ligands, for the possible interactions with amino acids.

The most spontaneous binding process was determined for the Cu-PLAG complex that contains two thiocyanate ions. The binding constants in the case of this complex compound range from 9.62 × 10^3^ to 1.86 × 10^6^ M^−1^, while the change in Gibbs free energy is between −22.52 and −36.77 kJ mol^−1^. The presence of thiocyanate ions allows the formation of multiple hydrogen bonds with the surrounding amino acids due to the presence of two electronegative atoms, although these interactions are much weaker than those formed between two Cu-PLAG complexes which leads to the high value of entropy change (404.98 kJ mol^−1^). These results show the importance of other ligands present in structure. It is important to mention that in a recent study that investigated the binding affinity of a series of pyridoxal–thiosemicarbazone complexes with various metals, the one containing Cu proved to bind more tightly to BSA than other compounds, with the change in free energy of binding around 30 kJ mol^−1^ [37]. Interactions involving thiocyanate ions are examined in detail in the following paragraph.

**Figure 8:**
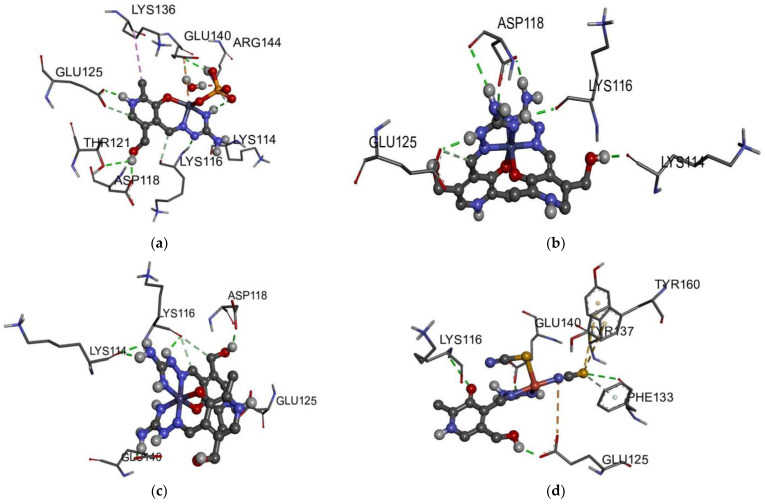
The best docking positions for the interactions between complexes and BSA: (**a**) Zn-PLAG, (**b**) Co-PLAG, (**c**) Fe-PLAG, and (**d**) Cu-PLAG (conventional hydrogen bonds—light green, hydrophobic—rose pink).

The binding energies and other important thermodynamic parameters for the interaction between BSA and investigated complexes are shown in Table 2. These values are −21.0 (Fe-PLAG), −23.1 (Cu-PLAG), −25.5 (Zn-PLAG), and −34.4 kJ mol^−1^ (Co-PLAG) with the binding constants between 2.1 × 10^−2^ and 88.4 µM. The differences between these values and the experimental ones are noticeable. One of the main reasons is the change in BSA structure when solvated by the water molecules and other ions of the phosphate buffer [50,51,52]. Moreover, complexes Cu-PLAG and Z-PLAG contain ligands loosely bound to the metal ion, and their exchange with the solvent molecules can be expected. The most stable confirmations of metal complexes–BSA structures are presented in Figure 8. The highest binding affinity was calculated for the Co-PLAG complex that formed several hydrogen bonds between ligands’ polar groups and surrounding amino acids (GLU125, ASP118, LYS116, and LYS114). This high number of hydrogen bonds is responsible for the highest affinity. The main contribution to this value comes from the sum of van der Waals, hydrogen bonding, and desolvation (−25.6 kJ mol^−1^), followed by the electrostatic interactions (−15.7 kJ mol^−1^). The presence of the sulfate group in the optimized structure of the Zn-PLAG complex is responsible for the second highest value of change in free energy of binding of this compound. As seen in Figure 8, the sulfate group interacts with GLU140 through hydrogen bonds. The PLAG ligand is also involved in forming hydrogen bonds with GLU125, THR121, and ASP118. The contribution from the weak interactions is higher than in the previously described complex (−31.1 kJ mol^−1^), although the contribution due to electrostatic interactions is much lower (−2.4 kJ mol^−1^). The calculated affinity of Fe-PLAG is much lower than for Co-PLAG, which is different than expected from the structural point of view. It can be postulated that the lack of charged groups allows the formation of conventional hydrogen bonds, π → π, and σ → π interactions. The thiocyanate ions of the Cu-PLAG complex are also involved in the stabilization interactions’ formation, for example, between sulfur and PHE133 (hydrogen bond)/TYR137 (σ → π). The interaction between the carbonyl oxygen of PLAG and LYS116 can also be described as a conventional hydrogen bond. The presence of charged ligands and the position of polar groups of PLAG proved to be important for the study of interactions but also reflect well the experimental range of binding energies, although the relative order is slightly different.

### 2.7. HSA Binding Affinity Analysis

**Figure 9:**
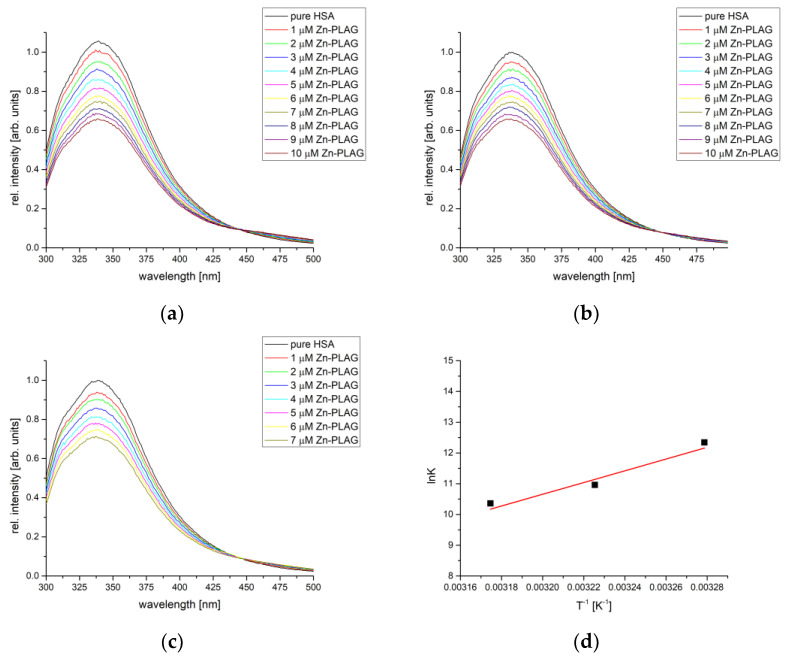
Fluorescence emission spectra of HSA for the titration with Zn-PLAG at (**a**) 32°, (**b**) 37°, (**c**) 42°, and (**d**) van’t Hoff plot for the binding process.

The binding affinity of obtained complexes was also examined towards HSA as a transport protein relevant to the human body. The temperature range was changed to reflect better temperatures common for humans (32–42 °C). The representative example of the HAS fluorescence measurements is shown in Figure 9, along with van’t Hoff’s plot. The binding constants, number of binding places, and thermodynamic parameters are given in Table 3. The decrease in the fluorescence intensity emission was also dependent on the concentration of complexes. On average, the binding constants were similar to the ones calculated for BSA. This result is expected since active pockets contain analogous amino acids. High correlation coefficients, between 0.990 and 0.998, were calculated for the dependency described by Equation (2). The number of binding places is 1, proving that the same type of complexes were formed between investigated compounds and HAS as previously shown for BSA.

Out of three newly obtained compounds, Zn-PLAG showed the narrowest range of the change in Gibbs free energy, between −28.65 and −29.07 kJ mol^−1,^ with the binding constants between 3.16 × 10^4^ and 2.29 × 10^5^ M^−1^. The formed interactions include hydrogen bonds and van der Waals interactions, as previously shown for other Zn complexes [53]. Two complexes containing two PLAG ligands have almost equal binding constants for all three temperatures (Co-PLAG: −23.34 (32 °C), −28.29 (37 °C), and −33.24 kJ mol^−1^ (42 °C); Fe-PLAG: −21.26 (32 °C), −27.81 (32 °C), and −34.36 kJ mol^−1^ (32 °C)). These values show that the most important for the binding of complexes are interactions between PLAG and amino acids, no matter what the central metal ion is. As shown in the previous section, these complexes have the same geometry with slight differences in bond lengths and angles. The binding affinity of Cu-PLAG is the lowest among the three complexes, except for the lowest temperature. Due to slightly different amino acid residues in the active pocket, this behavior is different from the HSA binding assay. It can be expected that due to the relative flexibility of thiocyanate ions in structure, the increase in temperature increases the system’s entropy and limits the number of interactions with the surrounding amino acids. The different behavior of these complexes is further examined by the molecular docking study.

### 2.8. DNA Binding Affinity

**Figure 10:**
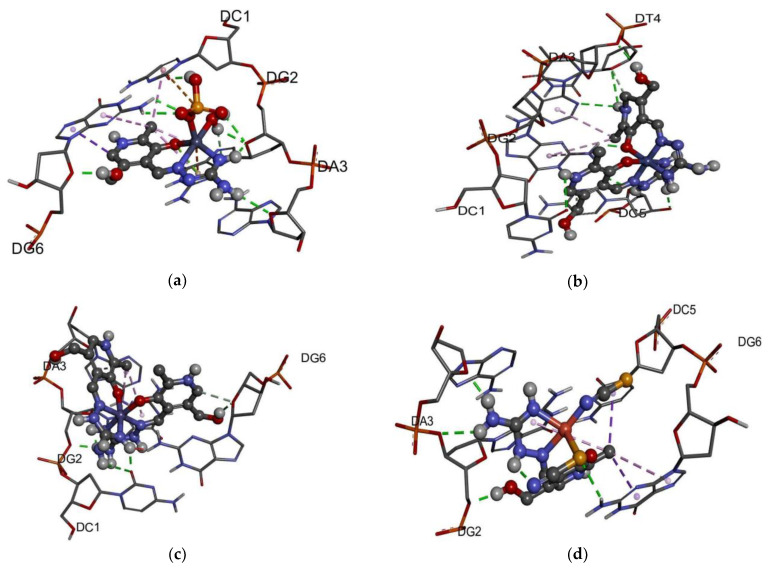
The best docking positions for the interactions between complexes and six-base-pair DNA: (**a**) Zn-PLAG, (**b**) Co-PLAG, (**c**) Fe-PLAG, and (**d**) Cu-PLAG (conventional hydrogen bonds–light green, hydrophobic–rose pink).

The DNA binding affinity of the obtained compounds was analyzed by the molecular docking towards a six-base pair DNA with the intercalation gap. These processes are observed experimentally in the anticancer regiments of several compounds [54]. The binding energy and other thermodynamic parameters of each complex are presented in Table 4, while Figure 10 shows the most stable docking positions. These complexes have a change in Gibbs free energy in the range between −25.7 (Fe-PLAG) and −30.0 kJ mol^−1^ (Zn-PLAG). Two positively charged complexes (Co-PLAG and Fe-PLAG) have interactions that are enhanced since DNA possesses a negatively charged phosphate backbone [55]. In the case of Zn-PLAG and Cu-PLAG, other present substituents also interact with the surrounding base pairs through different interactions. For all of the complexes, the main contribution comes from the weak interactions, including hydrogen bonds, van der Waals, attractive and repulsive interactions (between −36.0 (Zn-PLAG) and −32.3 kJ mol^−1^ (Co-PLAG and Fe-PLAG)). This result proves the importance of both small ligands, such as thiocyanate or sulfate, in the vicinity of the central metal ion and large ligands with many polar groups (PLAG). The electrostatic interactions are present for both types of ligands. The torsional change in Gibbs free energy is almost equal for all complexes, but higher in case of Zn-PLAG due to the relative flexibility of the ligands. It should be mentioned that the structure of complexes can potentially change when dissolved in polar solvents, and that the coordination sphere might contain solvent molecules instead of negatively charged anions [37]. The same reference concludes that the choice of metal ions and complex geometry significantly influence the interactions with biomolecules.

A closer look at the interactions with the six base pairs allows elucidation of the possible interactions (Figure 10). As expected, the presence of a sulfate group in the structure of Zn-PLAG is important for forming hydrogen bonds with the DC1 and DG2 [53]. The amino groups of PLAG interact with DA3, while hydroxymethyl oxygen forms hydrogen bonds with DG6. Water molecules in the inner sphere of Zn-PLAG also interact with DG6. The size of this complex was important for the intercalation into DNA structure [56]. The thiocyanate ligands within Cu-PLAG form hydrogen bonds with DG6 and the hydrophobic interaction with DG6 and DC5. These ligands contain two electronegative atoms within a small unit, which leads to the polarization that strengthens the interactions. The hydrogen bonds of the same type are formed with DA3 and DG2. The size of Fe-PLAG and Co-PLAG limits the possible interactions and inclusion of the complex, which explains the weaker binding affinity. Fe-PLAG formed hydrogen bonds with DA3, DG2, and DC1, and weaker interactions with DG6. The same applies to Co-PLAG. These promising values should be analyzed experimentally, as those results could explain the change in the structure of complexes when dissolved in water.

### 2.9. Cytotoxicity Analysis

The biological activity of investigated compounds was analyzed towards colorectal carcinoma (HCT116), melanoma (A375), breast cancer (MCF-7), and ovarian cancer (A2780) as the most common cancer types. The activities were determined by two assays, namely MTT and CV. Table 5 shows IC_50_ values with standard deviation, and dose–response curves are presented in Figures S8–S11.

Investigating the first-row transition metal complexes is important as some are non-toxic and very effective towards cancerous cell lines [57]. Based on the results from the previous table, it can be concluded that Zn-PLAG, Fe-PLAG, and Co-PLAG were not active in any of the cell lines due to the presence of investigated ligand system. Similar inactivity towards HCT116 cell lines was obtained in case of Fe(II)-phthalocyanine complex [58], while other complexes containing Fe(II) showed moderate toxicity towards HCT116 and MCF-7 cell lines [59,60]. Various compounds containing cobalt and Schiff bases were found to be active towards the MCF-7 cell line, with the enchantment of activity upon photoirradiation [61,62,63]. Complex Cu-PLAG showed moderate to high activity against investigated cell lines in a concentration-dependent manner. The lowest activity was obtained in the case of HCT116 cell lines ((81.5 ± 0.1) µM for MTT test and (97.4 ± 3.7) µM). The activity of the starting compound, CuSO_4,_ was shown to be almost completely inactive towards this cell line in the previous study [64], which leads to the conclusion that activity was increased due to the complexation with PLAG. The IC_50_ values are slightly lower when melanoma cells are concerned (81.5 ± 0.1) µM for MTT test and (97.4 ± 3.7) µM) for CV test. The complexation of three different thiosemicarbazone ligands with Cu(II) decreased IC_50_ value towards A375 to submicromolar and low micromolar levels, thus proving the importance of this metal ion [65,66]. These values are comparable to those obtained for a Cu complex with a similar ligand, pyridoxal-thiosemicarbazone [37]. The activity of Cu-PLAG significantly increased when MCF-7 and A2780 cells were concerned, and it is comparable to that of cisplatin ((33.6 ± 4.8) µM) measured by MTT assay [67]. The activity of diamine-based mixed ligand Cu(II) complexes towards A2780, but not to HCT116, was also found in the literature and it was proven that this activity was not due to different intracellular concentrations of complexes, as determined by the ICP-AES [68]. The results of a study including CuO nanoparticles proved that this compound could induce apoptosis in A2780 cells at a significant level [69]. The present ligands, besides PLAG, significantly influence the stability and reactivity of compounds, as proven throughout this article. The forthcoming in vivo studies will help elucidate the mechanism of action, which can only be assumed based on the obtained results. The IC_50_ values again show that the presence of Cu and specific geometries around it are promising in preparing novel anticancer agents.

## 3. Materials and Methods

### 3.1. Chemicals

All chemicals were obtained from commercial manufacturers and used without further purification.

### 3.2. Elemental Analysis

The elemental analyses (C, H, N, and S) of air-dried complexes were performed by the standard micromethods on an Elementar Vario El III. The samples were measured in triplicates and analyzed through oxidation in oxygen at 1200 °C. The gaseous oxides were then separated in a column with a carrier gas (helium). The mass percentage of each element was determined based on the intensity corresponding to different oxides.

### 3.3. Synthesis of Pyridoxylidene-Aminoguanidine, PLAG

An amount of 0.68 g (5 mmol) AG·H_2_CO_3_ was dissolved in 10 cm^3^ of H_2_O with heating, and to this solution, a warm solution of 1.0 g (5 mmol) PL∙HCl in 5 cm^3^ of H_2_O was added. To this mixture, 0.71 g (2.5 mmol) of Na_2_CO_3_·10H_2_O previously dissolved in 10 cm^3^ of H_2_O was added. The mixture was then left at room temperature. After 8 h, yellow crystals were filtered off and washed with EtOH and Et_2_O. Yield: 1.05 g (90%). Properties: Yellow powder; soluble in chloroform, dichloromethane, dimethylformamide, acetone, and acetonitrile, moderately soluble in methanol, ethanol, and water, insoluble in diethyl ether and toluene. Anal. Calcd for C_9_H_13_N_5_O_2_ (223.23): C, 48.42; H, 5.87; N, 31.37. Found: C, 48.22; H, 5.80; N, 31.47.

### 3.4. Synthesis of [Zn(PLAG)(SO_4_)(H_2_O)]H_2_O Complex

In 15 cm^3^ of warm H_2_O, 0.14 g (0.5 mmol) PLAG was dissolved, and a solution of 0.07 g (0.5 mmol) ZnSO_4_ in 5 cm^3^ H_2_O was added. The mixture was heated for about 5 min. After four days, pale orange single crystals were formed and washed with EtOH and Et_2_O. Yield: 0.13 g (79%). Properties: pale orange crystals; soluble in chloroform, dimethylsulfoxide, dimethylformamide, acetone, and acetonitrile, moderately soluble in methanol, ethanol, phosphate buffer saline, and water, insoluble in diethyl ether and toluene. Anal. Calcd for ZnC_9_H_17_N_5_O_8_ (420.70): C, 25.69; H, 4.07; N, 16.65; S, 7.62. Found: C, 25.49; H, 4.17; N, 16.72, S, 7.64.

### 3.5. Synthesis of [Co(PLAG)_2_]SO_4_^∙^2H_2_O Complex

A mixture of 0.11 g (0.5 mmol) PLAG and 0.07 g (0.25 mmol) CoSO_4_·7H_2_O was prepared and dissolved in 15 cm^3^ of H_2_O. Clear dark red solution was left at room temperature and taken out after ten days. The dark red single crystals were filtered and washed with MeOH. Yield: 0.06 g (36%). Properties: dark red crystals; soluble in chloroform, dimethylsulfoxide, dimethylformamide, acetone, and acetonitrile, moderately soluble in methanol, ethanol, phosphate buffer saline, and water, insoluble in diethyl ether and toluene. Anal. Calcd for CoC_18_H_28_N_10_O_10.75_S (647.49): C, 33.39; H, 4.36; N, 21.63; S, 4.95. Found: C, 32.93; H, 4.59; N, 21.38, S, 4.92.

### 3.6. Synthesis of [Fe(PLAG)_2_]SO_4_^∙^2H_2_O Complex

A mixture of 0.11 g (0.5 mmol) of PLAG and 0.10 g (0.25 mmol) FeSO_4_∙7H_2_O was mixed and dissolved in 7 cm^3^ of H_2_O. The mixture was heated to complete dissolution. After 20 h, dark red crystals were filtered and washed with EtOH and Et_2_O. Yield: 0.07 g (41%). Properties: dark red crystals; soluble in chloroform, dimethylsulfoxide, dimethylformamide, acetone, and acetonitrile, moderately soluble in methanol, ethanol, phosphate buffer saline, and water, insoluble in diethyl ether and toluene. Anal. Calcd for FeC_12_H_28_N_10_O_9.5_S (624.41): C, 34.63; H, 4.52; N, 22.43; S, 5.14. Found: C, 34.57; H, 4.56; N, 22.28, S, 5.00.

### 3.7. Synthesis of [Cu(PLAG)(NCS)_2_]_2_ Complex

The complex [Cu(PLAG)(NCS)_2_]_2_ was prepared as described in the literature [27]. Yield: 0.04 (86%). Properties: green rod-like crystals; soluble in chloroform, dimethylsulfoxide, dimethylformamide, acetone, and acetonitrile, moderately soluble in methanol, ethanol, phosphate buffer saline, and water, insoluble in diethyl ether and toluene. Anal. Calcd for CuC_11_H_13_N_7_O_2_S (370.88): C, 35.62; H, 13.10; N, 26.44; S, 8.65. Found: C, 35.15; H, 13.00; N, 26.58, S, 8.50.

### 3.8. X-ray Analysis

The X-ray measurements were performed on a single crystal of the complex mounted on glass fiber and examined at given temperatures. Bruker D8 Venture APEX diffractometer equipped with a Photon 100 area detector using graphite-monochromate Mo-Kα radiation [λ = 0.71073 Å] was used for these measurements. Based on the difference map, the location of hydrogen atoms was determined. The absorption corrections were performed with the SCALE3 ABSPACK algorithm implemented in the CrysAlisPro software (Rigaku, Cedar Park, TX, USA) [70]. Hydrogen atoms bound to carbon were initially positioned geometrically, while the hydrogen atoms for the coordinated water molecules were found through the difference map. All hydrogen positions and isotropic displacement parameters were refined in a separate cycle. Hydrogen positions were checked for feasibility by examination of the hydrogen-bonding network. Crystallographic data for the complexes were deposited in the Cambridge Crystallographic Data Centre (CCDC, 12 Union Road, Cambridge, UK; e-mail: depos-it@ccdc.cam.ac.uk); CDC deposition numbers are 2,268,971, 22,69,465, 2,271,164. Crystal data collection and structure refinement are given in Table 6.

### 3.9. Hirshfeld Analysis

Theoretical investigation of the intermolecular interactions within crystal structure is important for assessing the overall stability. Hirshfeld surface analysis is a method for identifying and quantifying the contacts between atoms of different unit cell constituents. CrystalExplorer program package [71] is used in this contribution for the analysis of crystal structure. Hirshfeld surface analysis is presented by a graph connecting two distances, namely the distance between the two nearest nuclei (de) and the distance between nuclei and the external surface (di) [72,73,74]. The distances are normalized and colored depending on the van der Walls radii separation. The red, white, and blue colors are used if the separation is shorter, equal, or longer than the van der Waals radii. The normalized distances in this contribution are between -0.6808 (red) and 1.1894 a.u. (blue). Fingerprint plots present different contact points between specific atoms and relative percentages within a total number of contacts.

### 3.10. Theoretical Analysis

The obtained crystallographic structures were optimized in the Gaussian 09 Program Package [75] (Gaussian 09, Revision C 01) without any geometrical constraints. The Global Hybrid Generalized Gradient Approximation (GGA) functional B3LYP [76] in conjunction with 6-31+G(d,p) basis set [77] for H, C, N, O, S, and Cl and LanL2DZ [78,79] for Fe, Co, Zn, and Cu was applied for the optimization. The same level of theory was successfully applied for theoretical analysis of similar compounds, structure optimization, and spectral assignation in the literature. The minima on the potential energy surface were obtained for all structures, as proven by the absence of imaginary frequencies. For the optimization of compounds, several metals’ spin states were analyzed, and the ones resembling the most crystallographic structure are described in this work and used for molecular docking. The intramolecular interactions governing stability were assessed by the Natural Bond Orbital Analysis (NBO) [80,81]. The stability of structures can also be investigated by the Quantum Theory of Atoms in Molecules (QTAIM) by calculating the electron density and Laplacian at the Bond Critical Points (BCP) and Ring Critical Points (RCP) [82,83]. The interactions can be divided into two groups based on the values of these parameters [84]. Closed-shell interactions (covalent bonds) are characterized by the electron density of 0.1 a.u. and large negative Laplacian. Open-shell interactions, including ionic bonds, hydrogen bonds, and van der Waals interaction, have electron density between 0.001 and 0.04 a.u. and a small positive Laplacian. The AIMAll package was used for these calculations [85].

Fukui functions are useful parameters for the determination of the electrophilic, nucleophilic, and radical reaction sites. In this contribution, these parameters were used to predict the possible sites for the interactions with species with unpaired electrons, such as transition metals. There are several methodologies proposed for the evaluation of the Fukui functions in the literature [86]. The Condensed Fukui functions (CFF) can be calculated for the specific atoms in the following way:

(1) $$f_{A}^{0}=\left[q_{N-1}^{A}-q_{N+1}^{A}\right]/2$$

In the previous equation, $q_{N-1}^{A}$ and $q_{N+1}^{A}$ represent charge on atom A in cationic and anionic species from the NBO analysis. When comparing CFFs, a more reactive site is the one with a higher value [87].

### 3.11. Stability Studies

The stability studies of complexes were performed in water and phosphate buffer saline for the final concentration of 10^−4^ M to verify to the use of these solutions in further biological studies. The stock solution of each complex was prepared in DMSO (10^−2^ M) and further diluted in mentioned media. The stability was followed by the UV-VIS spectrophotometer (Thermo Scientific UV-VIS Spectrophotometer) in the range between 300 and 800 nm, resolution 1 nm, integration time 0.20 s. Measurements were repeated 30 min, 24, 48, 72 h, and 2 months after the preparation of the solutions. These spectra are given in the Supplementary Information.

### 3.12. Spectrofluorimetric Measurements

The spectrofluorometric measurements were used to investigate the binding process of obtained compounds towards bovine serum albumin (BSA) and human serum albumin (HSA) on the Cary Eclipse MY2048CH03 instrument. The scan rate was 600 nm min^−1,^ and both slits were 5 nm. The excitation wavelengths were 295 nm and 280 nm for BSA and HSA, respectively. These energies are characteristics of the protein structure’s tryptophan and other fluorescent amino acid residues. The emission spectra were recorded between 310 and 500 nm. The concentration of proteins was held constant at 5 × 10^−6^ M in 1 M phosphate buffer saline (pH = 7.4). The concentration of complexes changed from 1 to 10 × 10^−6^ M, and the emission spectrum was recorded two minutes after the addition of compounds. The data were analyzed by the double log Stern–Volmer quenching equation (Equation (2)):

(2) $$\log\left(\frac{I_{0}-I}{I}\right)=\log K_{b}+n\log\left[Q\right]$$

In the previous equation, *I*_0_ and *I* are the fluorescent emission intensities of BSA/HSA without and with added metal complexes, respectively; *K*_b_ is the binding constant; *n* is the number of binding places; and [*Q*] is the concentration of quenchers (metal complexes).

The changes in enthalpy and entropy of the binding process were determined from the vant’t Hoff equation (Equation (3)) following the measurements at three temperatures (27, 32, and 37 °C):

(3) $$\ln K_{b}=-\frac{∆H_{b}}{RT}+\frac{∆S_{b}}{R}$$

### 3.13. Molecular Docking

The interactions between complexes and BSA/DNA were assessed by the molecular docking simulations. The AutoDock 4.2 [88] software was used for the examination of the binding affinity. The AMDock program [89] was utilized for localizing and examining the receptor’s pockets and binding sites. The crystal structures of the target were obtained from the RCSB Protein Data Bank in PDB format (1Z3F [90] and 40R0 [91]). Before docking, the targets were prepared by removing the co-crystalized ligand, water molecules, and cofactors in the AMDock program. The AutoDockTools graphical user interface [88] was employed to calculate the Kollman partial charges and add polar hydrogen atoms. The Lamarckian Genetic Algorithm (LGA) method [92] was used to perform protein–ligand flexible docking. The grid centers with dimensions 0.871 × 17.488 × 46.277 Å^3^ and 34.745 × 24.129 × 96.471 Å^3^ in -*x*, -*y*, and -*z* directions of the DNA and BSA were selected to cover the protein binding sites and allow the free movement of ligand. The contributions to the total energy were calculated by the following equation:

Δ**G**_bind_ *=* Δ**G**_vdw + hbond + desolv_ *+* Δ**G**_elec_ *+* Δ**G**_total_
*+* Δ**G**_tor_
*−* Δ**G**_unb_ (4)

where Δ**G**_bind_ is the estimated free energy of binding, the Δ**G**_vdw + hbond + desolv_ denotes the sum of the energies of dispersion and repulsion (Δ**G**_vdw_), hydrogen bond (Δ**G**_hbond_), and desolvation (**ΔG**_desolv_). The Δ**G**_total_ represents the final total internal energy, the Δ**G**_tor_ is torsional free energy, Δ**G**_unb_ is the unbound system’s energy, and Δ**G**_elec_ is electrostatic energy.

### 3.14. Cells

The following cell lines were used in this study: human cell lines (A375 melanoma, MCF-7 breast cancer, HCT116 colorectal carcinoma, and A2780 ovarian cancer. These cell lines were purchased from the American Type Culture Collection (Rockville, MD, USA). The A2780 cells were cultivated in DMEM, while all other cell lines were cultivated in the HEPES-buffered RPMI-1640 medium. The cell culture mediums were supplemented with penicillin (1000 units/mL), streptomycin (100 µg/mL), and 10% inactivated FBS. All cells were cultivated at 37 °C in a humidified atmosphere with 5% CO_2_.

### 3.15. Viability Assays (MTT and CV)

Two colorimetric viability assays were applied, namely 3-(4,5-dimethylthiazol-2-yl)-2,5-diphenyl-2H-tetrazolium bromide (MTT) and crystal-violet (CV) (Sigma Aldrich, St. Louis, MO, USA), according to the previous studies [67,93,94]. The cells were seeded overnight in 96-well plates: A2780 and MRC-5 8 × 10^3^ cells/well, HCT 116 6 × 10^3^ cells/well, A375 5 × 10^3^ cells/well, and MCF-7 at 10 × 10^3^ cells/well [67]. The standard solutions of complexes were prepared in DMSO. All cells were treated with the following concentrations of investigated compounds: 100, 50, 25, 12.5, 6.25, 3.125, and 1.56 µM. After 72 h of treatment, viability assays were conducted. For the CV assay, cells were fixed using 4% paraformaldehyde (PFA) for 20 min at room temperature after being washed once with PBS. Afterward, they were stained with 0.2% CV solution for 25 min. This procedure was followed by washing with double distilled water. The cells were left to dry overnight at room temperature. In the last step, 33% acetic acid was used to dissolve the remaining dye. The other test included incubating cells in MTT solution (0.5 mg L^−1^ in cell culture medium) for 20 min at 37 °C in a humid environment with 5% CO_2_. After one hour, the MTT solution was discarded, and the formed formazan was dissolved using DMSO. A plate reader (Spectromax, Molecular Devices, San Jose, CA, USA) was utilized for the absorption measurements at 570 and 670 (background) nm. The cell viability was calculated as the percentage compared to untreated cells, and the mean was obtained using a four-parametric logistic function. Each of the three independent experiments was performed in quadruplicate.

## 4. Conclusions

Three novel crystallographic structures containing pyridoxylidene-aminoguanidine were obtained and described. Obtained complexes, Zn(PLAG)(SO_4_)(H_2_O)]^.^H_2_O (Zn-PLAG), [Co(PLAG)_2_]SO_4_^.^2H_2_O (Co-PLAG), and [Fe(PLAG)_2_]SO_4_^.^2H_2_O) (Fe-PLAG) contained ligand in neutral form. Their structure was compared to that of Cu(PLAG)(NCS)_2_ (Cu-PLAG). The specific interactions, as assessed by the Hirshfeld surface analysis, and their contribution depended on the geometry and co-crystalized solvent molecules. Upon complexation, the structure of complexes was changed towards expected values for octahedral (Co-PLAG and Fe-PLAG) and square-pyramidal (Zn-PLAG and Cu-PLAG). The mean absolute error when optimized and crystallographic structures were compared was of the order of experimental error, except when significant changes in angle values were obtained. The binding affinity of complexes depended on the present ligands and their spatial distribution, with the Cu-PLAG complex having the highest affinity (between −22.52 and −36.77 kJ mol^−1^) towards BSA due to the presence of thiocyanate ligands, as shown by molecular docking. The calculated affinity towards DNA was the highest in the case of Zn-PLAG (−30 kJ mol^−1^). The cytotoxicity studies towards A375 melanoma, MCF-7 breast cancer, HC116 colorectal carcinoma, and A2780 ovarian cancer showed that Cu-PLAG is biologically the most active compound out of the analyzed ones. This result correlated with the experimentally and theoretically determined stability and protein binding affinity. Further studies towards more active compounds containing PLAG ligands are advised as choosing central metal ion, counterions, and other ligands allows fine-tuning of the geometry and biological activity.

## Figures and Tables

**Table 1:** Binding process’s parameters for the interaction between obtained complexes and BSA.

Compound	T [°C]	K_b_ [M^−1^]	n	R^2^	ΔH_b_ [kJ mol^−1^]	ΔS_b_ [J mol^−1^ K^−1^]	ΔG_b_ [kJ mol^−1^]
**Zn-PLAG**	27	5.80 × 10^4^	1.13	0.999	98.78	420.77	−27.45
	32	1.24 × 10^5^	1.09	0.997			−29.56
	35	2.08 × 10^5^	1.03	0.999			−31.66
**Co-PLAG**	27	1.86 × 10^4^	0.97	0.998	16.01	135.08	−24.51
	32	2.04 × 10^4^	0.98	0.999			−25.19
	35	2.29 × 10^4^	0.96	0.998			−25.86
**Fe-PLAG**	27	3.18 × 10^4^	1.12	0.998	154.38	600.66	−25.82
	32	8.32 × 10^4^	1.10	0.990			−28.82
	35	2.34 × 10^5^	1.04	0.999			−31.82
**Cu-PLAG**	27	9.63 × 10^3^	1.13	0.998	404.98	1425.02	−22.52
	32	8.97 × 10^4^	1.10	0.998			−29.65
	35	1.82 × 10^6^	0.98	0.993			−36.77

**Table 2:** The important thermodynamic parameters for the best docking conformation of investigated complexes with BSA (PDB ID:4OR0).

Compound	ΔG_bind_	K_i_ (µM)	ΔG_vdw + hbond + desolv_	ΔG_elec_	ΔG_total_	ΔG_tor_	ΔG_unb_
**Zn-PLAG-BSA**	−25.5	33.4	−31.1	−2.4	−13.1	8.0	−13.1
**Co-PLAG-BSA**	−34.4	9.4 × 10^−1^	−25.6	−15.7	2.6	6.9	2.6
**Fe-PLAG-BSA**	−21.0	88.4	−28.3	−1.7	1.4	6.9	1.4
**Cu-PLAG-BSA**	−23.1	2.1 × 10^−2^	−26.8	−1.0	−5.1	6.9	−5.1

**Table 3:** Binding process’s parameters for the interaction between obtained complexes and HSA.

Compound	T [°C]	K_b_ [M^−1^]	n	R^2^	ΔH_b_ [kJ mol^−1^]	ΔS_b_ [J mol^−1^ K^−1^]	ΔG_b_ [kJ mol^−1^]
**Zn-PLAG**	32	3.16 × 10^4^	1.11	0.998	−158.66	−419.11	−28.65
	37	4.78 × 10^4^	0.99	0.998			−28.86
	42	2.29 × 10^5^	0.95	0.995			−29.07
**Co-PLAG**	32	9.84 × 10^3^	1.05	0.999	278.64	990.11	−23.34
	37	5.94 × 10^4^	1.08	0.995			−28.29
	42	3.22 × 10^5^	1.16	0.997			−33.24
**Fe-PLAG**	32	4.37 × 10^3^	1.09	0.992	378.28	1309.95	−21.26
	37	4.88 × 10^4^	0.90	0.990			−27.81
	42	4.97 × 10^5^	1.12	0.992			−34.36
**Cu-PLAG**	32	7.59 × 10^3^	0.91	0.990	128.56	495.17	−22.47
	37	1.38 × 10^4^	0.90	0.995			−24.94
	42	3.80 × 10^4^	0.98	0.996			−27.42

**Table 4:** The important thermodynamic parameters for the best docking conformation of investigated complexes with DNA (PDB ID:1Z3F).

Compound	ΔG_bind_	K_i_ (µM)	ΔG_vdw + hbond + desolv_	ΔG_elec_	ΔG_total_	ΔG_tor_	ΔG_unb_
**Zn-PLAG-DNA**	−30.0	5.6	−36.0	−2.1	−6.4	8.0	−6.4
**Co-PLAG-DNA**	−28.2	11.5	−32.3	−2.8	2.3	6.9	2.3
**Fe-PLAG-DNA**	−25.7	31.6	−32.3	−0.3	1.5	6.9	1.5
**Cu-PLAG-DNA**	−28.0	12.5	−33.5	−1.4	−5.4	6.9	−5.0

**Table 5:** IC_50_ values (in μM) for the cytotoxicity studies on the investigated complexes.

Cell Line		Assay	Zn-PLAG	Fe-PLAG	Co-PLAG	Cu-PLAG
HCT116	Colorectal carcinoma	MTT	>100	>100	>100	81.5 ± 0.1
		CV	>100	>100	>100	97.4 ± 3.7
A375	melanoma	MTT	>100	>100	>100	73.6 ± 2.0
		CV	>100	>100	>100	79.8 ± 0.8
MCF-7	Breast cancer	MTT	>100	>100	>100	48.3 ± 1.5
		CV	>100	>100	>100	65.4 ± 4.8
A2780	Ovarian cancer	MTT	>100	>100	>100	32.5 ± 2.6
		CV	>100	>100	>100	38.5 ± 2.1

**Table 6:** Crystallographic data of the newly obtained structure of Zn-PLAG, Fe-PLAG, and Cu-PLAG.

Empirical Formula	Zn-PLAG C_9_H_17_N_5_O_8_SZn	Co-PLAG C_18_H_28_CoN_10_O_10.75_S	Fe-PLAG C_18_H_28_FeN_10_O_9.5_S
Molecular mass	420.70	647.49	624.41
Crystal system	Triclinic	Triclinic	Monoclinic
Space group	P-1	P-1	I 1 2/a 1
a (A)	9.1031 (6)	11.9844 (3)	10.9269 (7)
b (A)	9.1317 (5)	12.5247 (4)	15.2428 (11)
c (A)	10.0627 (4)	12.6657 (4)	29.873 (2)
α (°)	85.167 (4)	101.946 (3)	90
β (°)	80.073 (4)	113.517 (3)	91.633 (6)
Γ (°)	65.473 (6)	110.863 (3)	90
V (A^3^)	749.53 (8)	1487.60 (9)	4973.6 (6)
Z	2	2	2
F(000)	432.0	685.0	6.309
Dx, g cm^−3^	1.864	1.471	1.668
Theta (max)	87.700	80.135	80.195
hkl Ranges	11, 11, 12	15, 15, 16	13, 19, 38
T (°C)	−100.00 (10)	−150.00 (10)	−150.00 (10)

## Data Availability

Not applicable.

## Supplementary Materials

The following supporting information can be downloaded at: https://www.mdpi.com/article/10.3390/ijms241914745/s1.
