# Supplementary material for: Synthesis, Structural Characterization, Cytotoxicity, and Protein/DNA Binding Properties of Pyridoxylidene-Aminoguanidine-Metal (Fe, Co, Zn, Cu) Complexes
Source: Int J Mol Sci. 2023 Sep 29;24(19):14745. doi: 10.3390/ijms241914745 (PMC10573062; doi:10.3390/ijms241914745)
Supplement: Supplementary file 1 [file ijms-24-14745-s001.zip › ijms-2614812-supplementary.pdf]

Supplementary information for:

Synthesis, structural characterization, cytotoxicity, protein, RNA, and DNA binding properties of pyridoxylidene-aminoguanidine-metal (Fe, Co, Zn, Cu) complexes

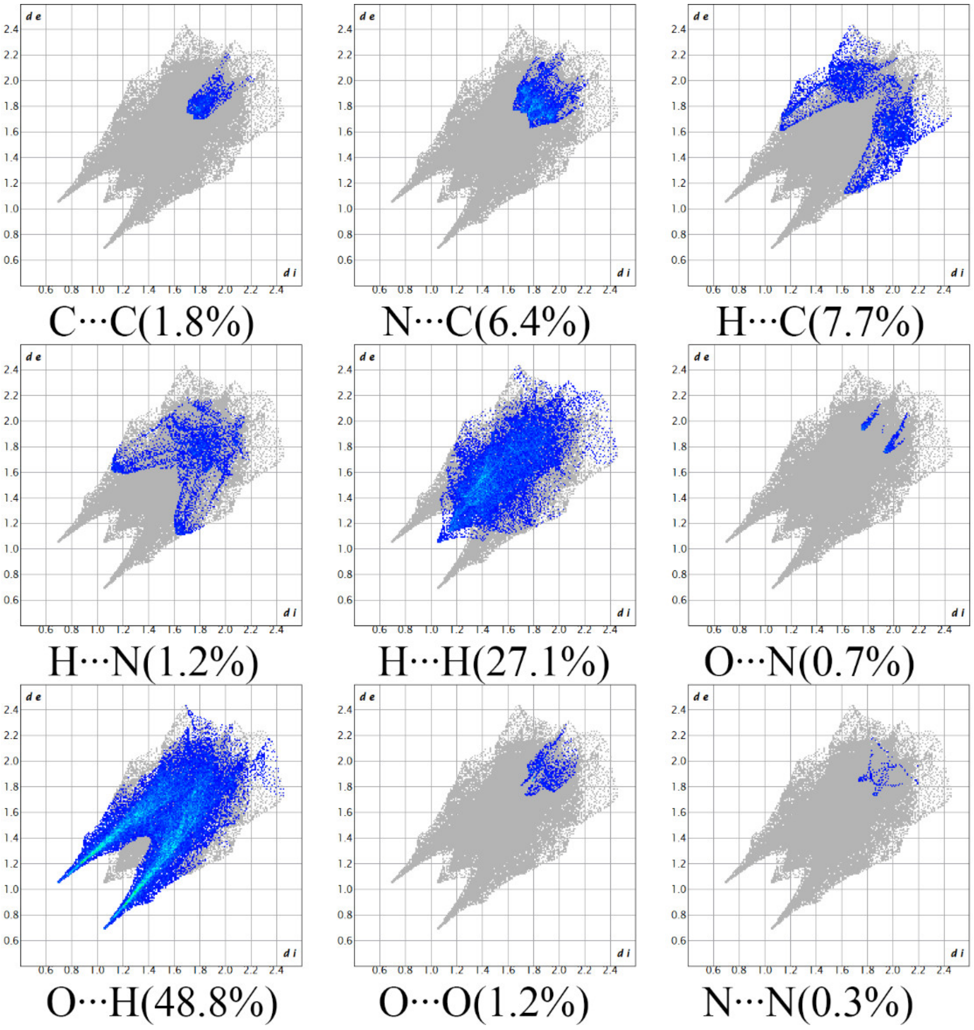

Figure S1. The fingerprint plots of the selected contacts within PLAG structure

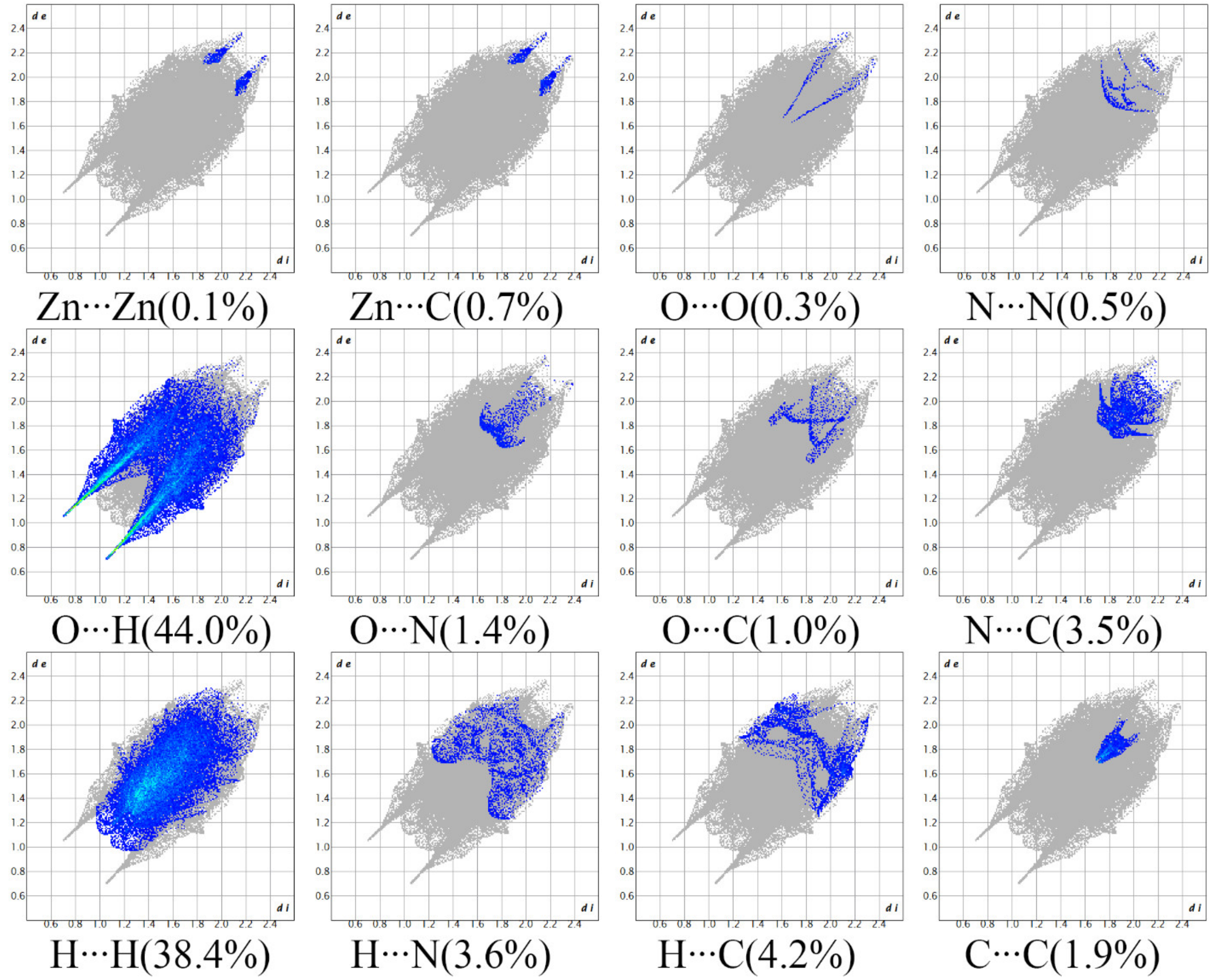

Figure S2. The fingerprint plots of the selected contacts within Zn-PLAG structure

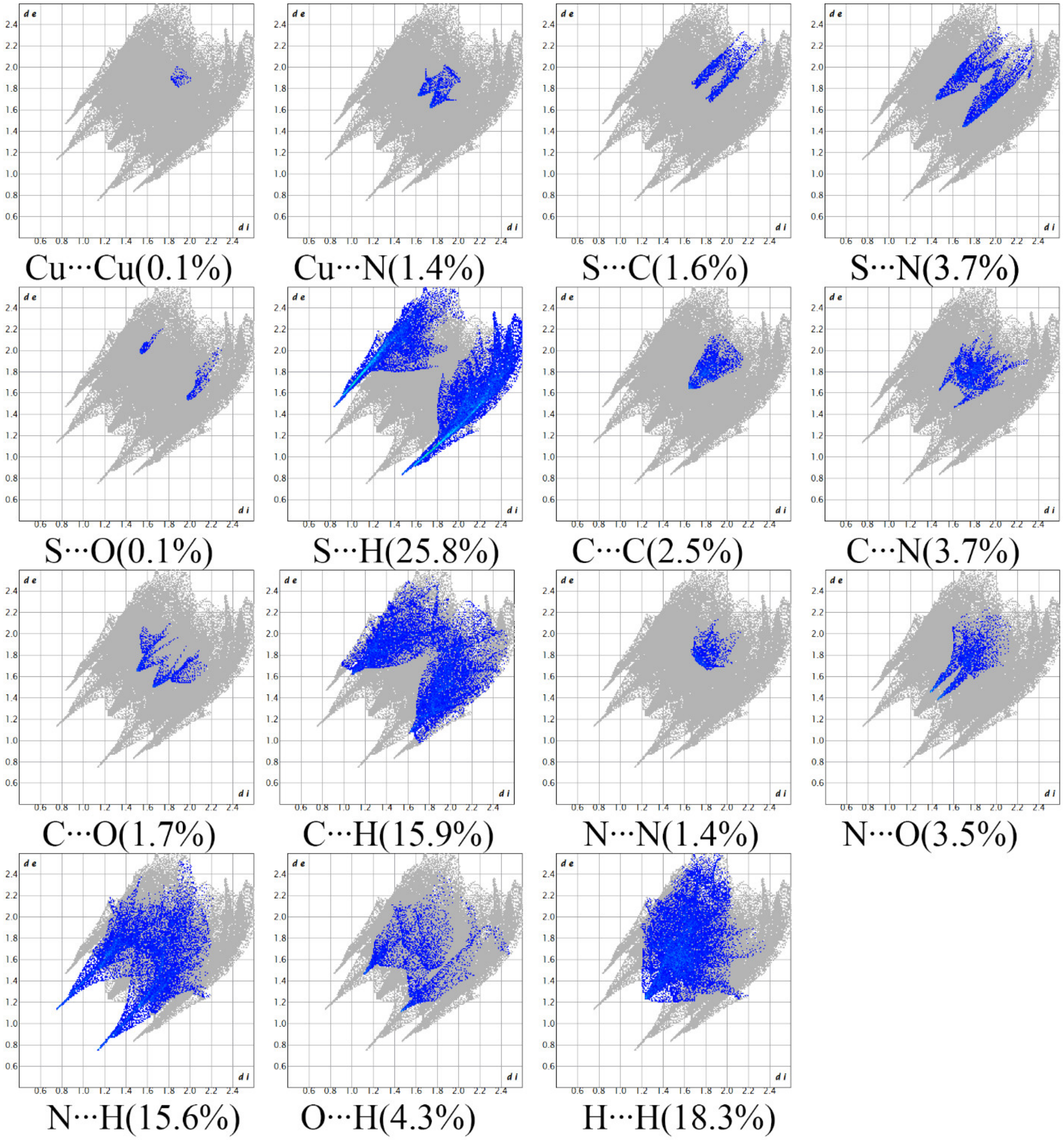

Figure S3. The fingerprint plots of the selected contacts within Cu-PLAG structure

Table S1. Experimental and theoretical (at B3LYP/6-311++G(d,p) level of theory) bond lengths of PLAG.

Bond	Experimental	Theoretical
C1-C2	1.37	1.37
C2-C3	1.41	1.42
C2-C7	1.51	1.52
C7-O	1.41	1.42
C3-C4	1.43	1.46
C3-C8	1.46	1.43
C4-C5	1.42	1.46
C4-O	1.29	1.24
C5-C6	1.48	1.49
C5-N1	1.33	1.33
N1-C1	1.35	1.37
C8-N2	1.28	1.30
N2-N3	1.38	1.39
N3-C9	1.34	1.33
C9-N4	1.31	1.33
C9-N5	1.31	1.34
MAE [Å]		0.02

Table S2. Experimental and theoretical (at B3LYP/6-311++G(d,p) level of theory) bond angles of PLAG.

C1-C2-C3	118.3	119.3
C1-C2-C7	118.3	118.1
C2-C7-O	112.7	113.5
C7-C2-C3	123.5	122.6
C2-C3-C4	120.3	120.9
C2-C3-C8	124.7	126.3
C8-C3-C4	114.9	112.8
C3-C4-C5	117.6	115.6
C3-C4-O	122.9	124.7
O-C4-C5	119.5	119.6
C4-C5-N1	118.8	118.8
C4-C5-C6	122.4	120.3
C6-C5-N1	118.7	120.9
C5-N1-C1	124.4	125.9
C3-C8-N2	123.5	126.6
C8-N2-N3	115.4	113.9
N2-N3-C9	117.2	119.3

N3-C9-N4	119.9	118.9
N3-C9-N5	119.1	120.1
N4-C9-N5	121.0	121.0
MAE [°]		1.3

Table S3. Second order perturbation theory stabilization energies (in kJ mol^{-1}) of PLAG.

Donor	Acceptor	Stabilization energy
π(Oc-C4)	π*(C5-N1)	43.4
π(Oc-C4)	π*(C3-C4)	8.9
σ(N2-N3)	σ*(C3-C8)	12.0
π(N2-C8)	π(N4-C9)	33.0
π(N3-C9)	π*(C8-N2)	45.0
π(N1-C5)	π*(C1-C2)	61.4
σ*(C3-C8)	σ(N2-N3)	19.1
π(C1-C2)	π*(N1-C5)	68.9
LP(Oc)	π*(C4-C5)	59.9
LP($O_{hydroxymethyl}$)	σ*(C2-C6)	37.2
LP(N4)	π*(C9-N3)	377.8
LP(N5)	π*(C9-N3)	343.3
LP(N2)	π*(C8-C3)	6.3

Table S4. CFFs for the interactions of PLAG's atoms with radical species

Atom	CFF value
C5	0.133
Oc	0.126
N2	0.090
N4	0.084
C3	0.084
C1	0.072
N3	0.066
C6-H	0.050
N1	0.036
N1-H	0.031
C1-H	0.030
C6-H	0.028
C8-H	0.028
C2	0.028
N3-H	0.025
C7-H	0.022
C8	0.022
C7-H	0.020
O-H	0.019
N5	0.016
N5-H	0.016

N5-H	0.010
Ohy	0.007
C6-H	0.007
N4-H	0.000
C9	-0.004
C7	-0.009
N4-H	-0.009
C4	-0.013
C6	-0.015

Table S5. Experimental and theoretical (at B3LYP/6-31+G(d,p)(H,C,N,O,S)/LanL2DZ(Zn) level of theory) bond lengths of Zn-PLAG.

Zn-O4	2.06	2.08
O4-S2	1.54	1.54
S2-O5	1.48	1.49
S2-O12	1.46	1.46
S2-O11	1.65	1.66
Zn-O6	2.20	2.23
Zn-O3	2.02	2.04
Zn-N13	2.14	2.17
Zn-N17	2.07	2.1
O3-C23	1.30	2.35
C23-C26	1.42	1.41
C23-C28	1.46	1.44
C26-C32	1.50	1.45
C26-N14	1.36	1.38
N14-C36	1.35	1.34
C36-C27	1.38	1.39
C27-C29	1.52	1.55
C29-O9	1.43	1.47
C27-C28	1.43	1.43
C28-C24	1.44	1.45
C24-N13	1.31	1.38
N13-N16	1.34	1.35
N16-C22	1.38	1.39
C22-N17	1.32	1.33
C22-N19	1.38	1.38
MAE [Å]		0.01

Table S6. Experimental and theoretical (at B3LYP/6-31+G(d,p)(H,C,N,O,S)/LanL2DZ(Zn) level of theory) bond angles of Zn-PLAG.

O4-Zn-O3	118.5	120.8
O4-Zn-O6	79.7	81.3
O4-Zn-N13	101.3	103.3
O4-Zn-N17	100.7	102.7
O3-Zn-O6	89.7	91.5
O3-Zn-N13	85.8	87.5
O3-Zn-N17	139.3	142.1
O6-Zn-N13	175.3	178.8
O6-Zn-N17	108.8	111.0
N13-Zn-N17	75.6	77.1
Zn-O4-S2	126.8	129.4
O4-S2-O5	110.3	112.5
O4-S2-O11	103.8	105.9
O4-S2-O12	111.8	114.1
O5-S2-O11	106.8	108.9
O5-S2-O12	118.9	121.3
Zn-O3-C23	128.9	131.4
O3-C23-C26	116.5	118.8
O3-C23-C28	125.9	128.4
C23-C26-C32	121.9	124.4
C23-C26-N14	120.0	122.4
C26-N14-C36	123.7	126.2
N14-C36-C27	119.8	122.2
C36-C27-C29	117.0	119.3
C27-C29-O9	113.7	116.0
C29-C27-C28	122.5	124.9
C27-C28-C23	118.3	120.6
C27-C28-C24	117.8	120.2
C28-C24-N13	122.7	125.2
C24-N13-Zn	128.2	130.8
Zn-N13-N16	112.1	114.3
N13-N16-C22	110.6	112.8
C16-C22-N19	112.4	114.7
C16-C22-N17	123.7	126.2
C22-N17-Zn	108.8	110.9
MAE [°]		2.3

Table S7. Experimental and theoretical (at B3LYP/6-31+G(d,p)(H,C,N,O)/LanL2DZ(Co) level of theory) bond lengths of Co-PLAG.

Co-O2	1.91	1.91
Co-O3	1.94	1.90
Co-N6	1.93	1.89
Co-N10	1.94	1.88
Co-N12	1.92	1.89
Co-N14	1.91	1.88
O2-C34	1.33	1.30
C34-C35	1.38	1.41
C35-N18	1.39	1.34
N18-C53	1.46	1.37
C53-C55	1.51	1.37
C55-C56	1.50	1.50
C56-O32	1.44	1.40
C55-C38	1.37	1.44
C38-C34	1.48	1.43
C38-C39	1.45	1.43
C39-N14	1.30	1.30
N14-N9	1.37	1.37
N9-C31	1.34	1.37
C31-N10	1.33	1.31
C31-N22	1.38	1.34
C3-C30	1.28	1.30
C30-C45	1.44	1.42
C45-C49	1.49	1.48
C45-N20	1.34	1.34
N20-C36	1.37	1.34
C36-C28	1.38	1.37
C28-C46	1.52	1.52
C28-C29	1.43	1.42
C29-C30	1.45	1.42
C29-C25	1.44	1.44
C25-N6	1.30	1.29
N6-N7	1.38	1.37
N7-C27	1.38	1.37
C27-N12	1.29	1.30
C27-N15	1.37	1.33
MAE [°]		0.03

Table S8. Experimental and theoretical (at B3LYP/6-31+G(d,p)(H,C,N,O)/LanL2DZ(Co) level of theory) bond angles of Co-PLAG.

O2-Co-O3	85.7	87.5
O2-Co-N6	86.0	89.5
O2-Co-N10	173.7	177.3
O2-Co-N12	93.1	90.7
O2-Co-N14	94.6	95.4
O3-Co-N6	92.4	93.6
O3-Co-N10	89.1	91.8
O3-Co-N12	174.5	175.8
O3-Co-N14	90.1	89.0
N6-Co-N10	97.7	93.1
N6-Co-N12	82.2	82.6
N6-Co-N14	177.5	174.6
N10-Co-N12	92.4	90.1
N10-Co-N14	81.8	82.0
N12-Co-N14	95.3	95.0
Co-O2-C34	122.1	124.0
O2-C34-C35	120.2	115.9
O2-C34-C38	121.9	125.4
C34-C35-C41	123.5	122.2
C34-C35-N18	119.3	119.3
C41-C35-N18	117.2	118.6
C35-N18-C53	117.4	123.0
N18-C53-C55	108.9	119.9
C53-C55-C56	115.2	117.8
C53-C55-C38	117.6	118.9
C55-C56-O32	107.5	111.5
C56-C55-C38	127.2	123.3
C55-C38-C34	119.1	118.4
C55-C38-C39	120.5	118.9
C38-C39-N14	125.8	124.6
C39-N14-Co	126.0	126.2
Co-N14-N9	115.8	116.2
C39-N14-N9	118.2	117.6
N14-N9-C31	110.4	109.3
N9-C31-N10	121.6	119.5
N9-C31-N22	116.3	116.7
MAE [°]		2.2

Table S9. Experimental and theoretical (at B3LYP/6-31+G(d,p)(H,C,N,O)/LanL2DZ(Fe) level of theory) bond lengths of Fe-PLAG.

Fe-O2	1.98	1.94
Fe-O3	1.98	1.96
Fe-N6	1.92	2.20
Fe-N10	2.01	2.02
Fe-N9	1.92	2.17
Fe-N18	2.01	2.04
O2-C33	1.30	1.30
C3-C43	1.41	1.42
C43-C53	1.38	1.50
C43-N16	1.37	1.33
N16-C44	1.37	1.36
C44-C42	1.37	1.38
C42-C57	1.51	1.52
C57-O23	1.43	1.40
C42-C34	1.45	1.41
C34-C33	1.47	1.41
C34-C39	1.41	1.45
C39-N6	1.34	1.29
N6-N12	1.40	1.37
N12-C41	1.37	1.36
C41-N10	1.29	1.30
C41-N20	1.40	1.35
O3-C30	1.29	1.31
C30-C32	1.41	1.41
C32-C49	1.49	1.49
C32-N14	1.39	1.34
N14-C37	1.37	1.36
C37-C35	1.37	1.36
C35-C46	1.50	1.51
C46-O4	1.44	1.44
C35-C36	1.45	1.42
C36-C28	1.40	1.46
C28-N9	1.33	1.29
N9-N7	1.40	1.36
N7-C31	1.37	1.36
C31-N18	1.30	1.31
C31-N25	1.40	1.33
MAE [Å]		0.04

Table S10. Experimental and theoretical (at B3LYP/6-31+G(d,p)(H,C,N,O)/LanL2DZ(Fe) level of theory) bond angles of Fe-PLAG.

O2-Fe-O3	90.32	86.45
O2-Fe-N6	89.81	81.05
O2-Fe-N9	91.40	122.51
O2-Fe-N10	170.83	150.94
O2-Fe-N18	89.30	91.92
O3-Fe-N6	91.02	118.44
O3-Fe-N9	90.03	81.32
O3-Fe-N10	90.19	92.78
O3-Fe-N18	90.19	150.48
N6-Fe-N9	178.39	151.73
N6-Fe-N10	81.03	73.82
N6-Fe-N18	97.68	90.28
N10-Fe-N18	91.58	102.40
Fe-O2-C33	126.93	133.20
O2-C33-C43	117.90	117.44
C33-C43-C53	122.62	120.08
C33-C43-N16	120.06	120.40
C53-C43-N16	117.30	119.52
C43-N16-C44	123.22	121.66
N16-C44-C42	120.02	121.86
C44-C42-C57	119.10	119.33
C42-C57-O23	110.41	109.77
C57-C42-C34	120.08	122.31
C44-C42-C34	120.81	118.35
C42-C34-C39	120.14	118.83
C42-C34-C33	117.80	119.39
C34-C39-N6	124.14	122.12
C39-N6-Fe	128.41	129.10
C39-N6-N12	115.60	117.24
N6-N12-C41	113.83	114.23
N12-C41-N10	118.08	117.95
C41-N10-Fe	112.97	120.66
C12-C41-N20	114.76	116.74
MAE [°]		7.5

Table S11. Experimental and theoretical (at B3LYP/6-31+G(d,p)(H,C,N,O)/LanL2DZ(Cu) level of theory) bond lengths of Cu-PLAG.

Cu-S2	2.41	2.88
S2-C3	1.67	1.65
C3-N4	1.17	1.17
Cu-N21	1.89	1.97
N21-C22	1.20	1.16
C22-S23	1.59	1.64
Cu-N16	2.10	1.95
Cu-N13	1.99	1.96
Cu-O5	2.17	1.92
N16-C15	1.29	1.29
C15-N17	1.35	1.35
C15-N14	1.41	1.37
N14-N13	1.39	1.37
N13-C12	1.30	1.28
C12-C11	1.44	1.45
C11-C6	1.44	1.43
C6-O5	1.26	1.30
C6-C7	1.45	1.43
C7-C20	1.49	1.49
C7-N8	1.33	1.33
N8-C9	1.37	1.36
C9-C10	1.38	1.37
C10-C11	1.43	1.41
C10-C18	1.52	1.51
C18-O19	1.42	1.43
MAE [Å]		0.05

Table S12. Experimental and theoretical (at B3LYP/6-31+G(d,p)(H,C,N,O,S)/LanL2DZ(Cu) level of theory) bond angles of Cu-PLAG.

S2-Cu-N21	89.76	95.03
S2-Cu-N16	121.00	96.71
S2-Cu-N13	95.04	92.27
S2-Cu-O5	102.11	90.83
N21-Cu-N16	121.00	93.07
N21-Cu-N13	175.21	171.56
N21-Cu-O5	94.87	93.42
N16-Cu-N13	77.43	81.85
N16-Cu-O5	134.02	169.58

N13-Cu-O5	84.06	90.71
Cu-S2-C3	95.45	93.85
S2-C3-N4	175.71	177.81
Cu-N21-C22	136.57	149.79
N21-C22-S23	174.46	178.82
Cu-N16-C15	111.53	113.62
N16-C15-N17	128.25	127.18
N16-C15-N14	117.79	117.69
C15-N14-N13	112.80	115.42
N14-N13-Cu	112.79	111.15
N14-N13-C12	116.33	118.40
N13-C12-C11	124.92	122.17
C12-C11-C6	120.44	122.07
C12-C11-C10	119.09	118.70
C11-C6-O5	125.89	126.05
C6-O5-Cu	124.88	127.40
C11-C6-C7	116.74	117.55
O5-C6-C7	117.36	116.39
C6-C7-C20	120.61	122.11
C20-C7-N8	120.69	118.88
C7-N8-C9	125.33	124.87
N8-C9-C10	119.49	119.16
C9-C10-C18	118.17	117.13
C9-C10-C11	119.32	119.99
C10-C18-O19	113.07	112.33
C18-C10-C11	122.49	122.79
MAE [°]		4.9

Table S13. Second order perturbation theory stabilization energies (in kJ mol^{-1}) of PLAG metal complexes.

Donor	Acceptor	Stabilization energy
LP($O_{sulfate}$)	LP*(Zn)	91
LP($O_{sulfate}$)	σ*(S-O)	48-119
LP($O_{phenolic}$)	LP*(Zn)	108
LP($N_{hydrazine}$)	LP*(Zn)	156
LP(N_{imino})	LP*(Zn)	164
LP(O_{water})	LP*(Zn)	80
LP($O_{phenolic}$)	σ*(Co-O)	64
LP($N_{hydrazine}$)	σ*(Co-N)	45
LP(N_{imino})	σ*(Co-O)	12
LP(N_{imino})	σ*(Co-N)	5

σ(Co-N)	σ*(Co-O)	42
LP($O_{phenolic}$)	σ*(Fe-O)	82
LP($N_{hydrazine}$)	σ*(Fe-N)	9
π(N_{imino}-C)	σ*(Fe-N)	18
σ*(Fe-N)	π*(N_{amino}-C)	66
LP(S)	LP*(Cu)	8
LP(S)	π*(C-N)	156
LP(N)	π*(C-S)	59
LP($O_{phenolic}$)	LP*(Cu)	103
LP($N_{hydrazine}$)	LP*(Cu)	179
LP($N_{thiocyanate}$)	LP*(Cu)	167

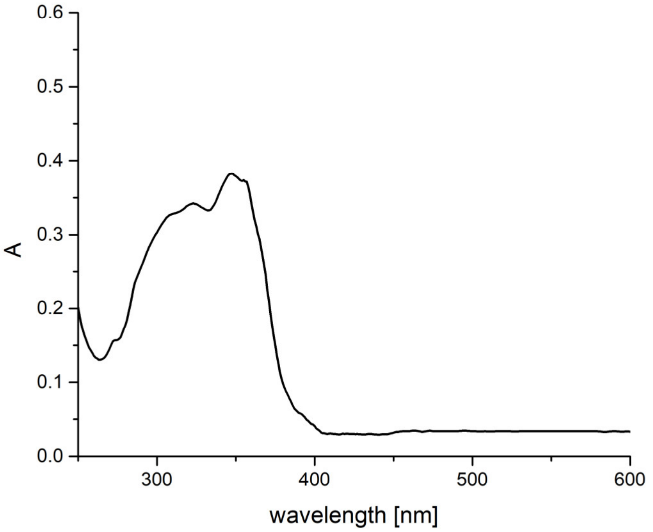

Figure S5. Electronic spectrum of PLAG in water.

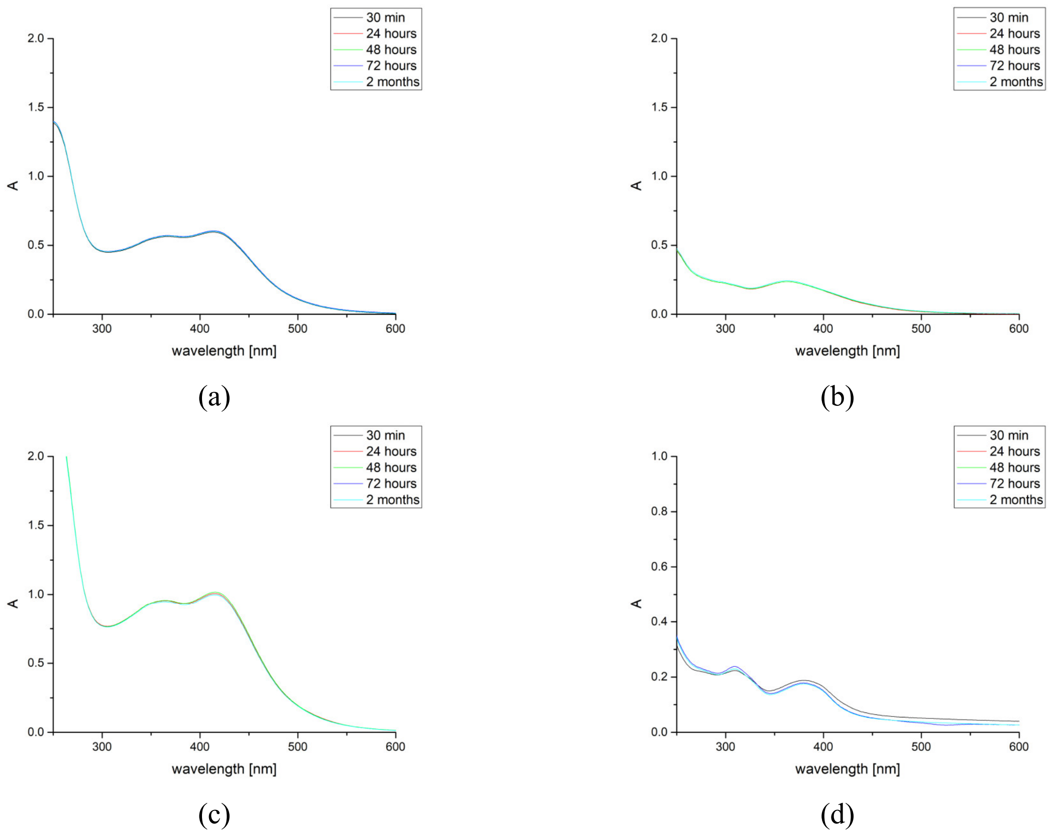

Figure S6. UV-VIS spectra for the stability studies in water:DMSO (1:10^{-4} (v:v)) solution of (a) Zn-PLAG, (b) Co-PLAG, (c) Fe-PLAG, and (d) Cu-PLAG

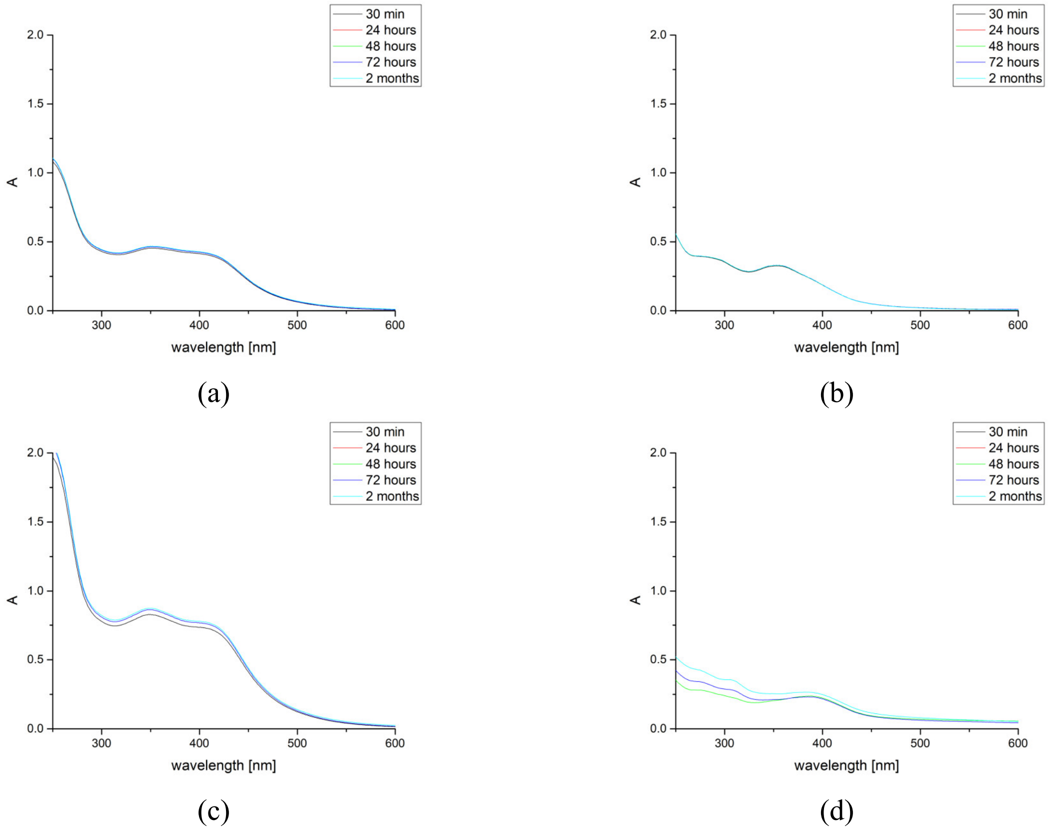

Figure S7. UV-VIS spectra for the stability studies in phosphate buffer saline:DMSO (1:10^{-4} (v:v)) solution of (a) Zn-PLAG, (b) Co-PLAG, (c) Fe-PLAG, and (d) Cu-PLAG

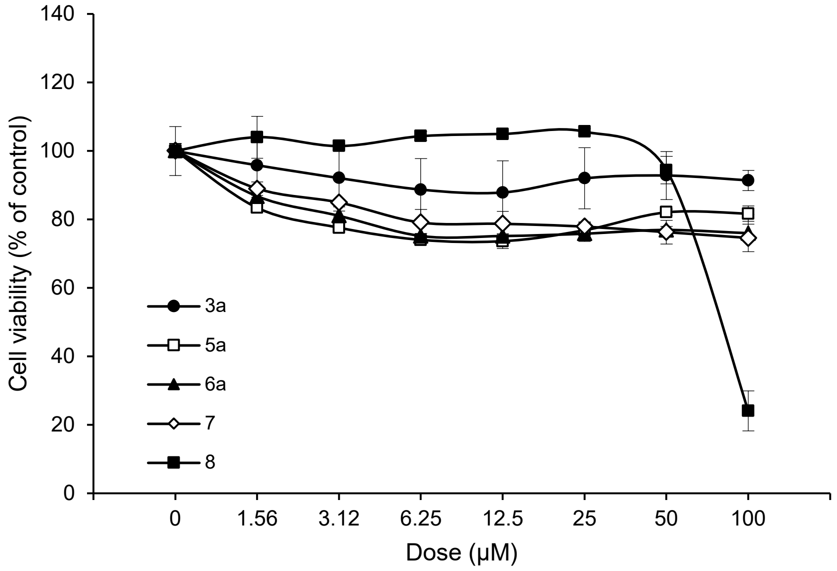

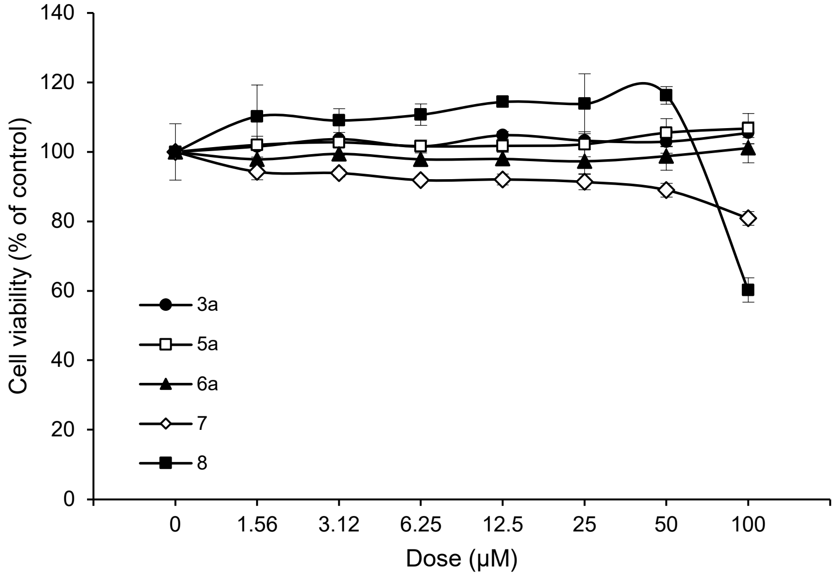

Figure S8. The dose-response curve for the activity towards HCT116 measured by MTT (upper) and CV (lower) tests (Zn-PLAG – 3a, Co-PLAG – 6a, Fe-PLAG – 5a, Cu-PLAG – 8).

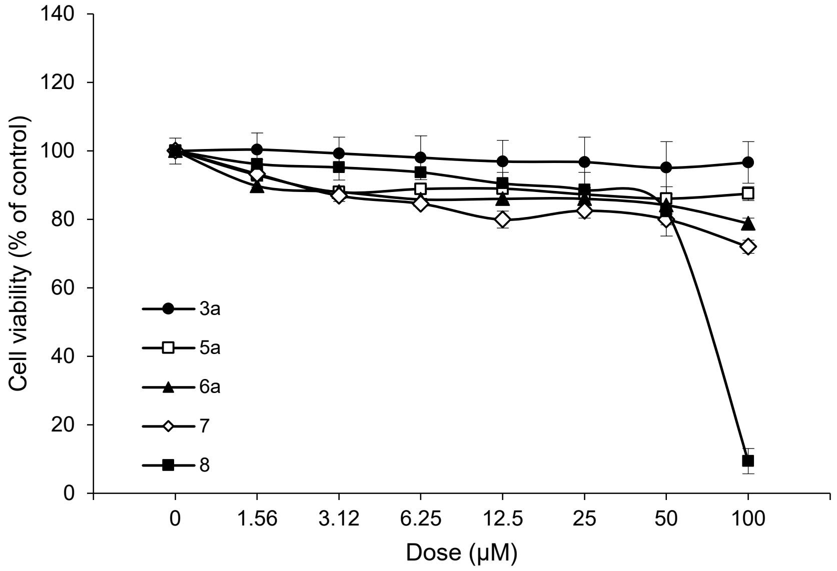

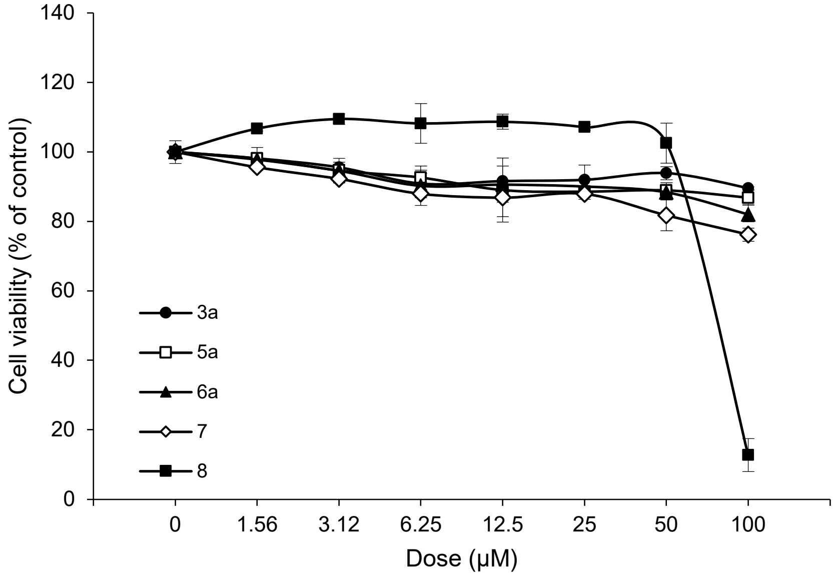

Figure S9. The dose-response curve for the activity towards A375 measured by MTT (upper) and CV (lower) tests (Zn-PLAG – 3a, Co-PLAG – 6a, Fe-PLAG – 5a, Cu-PLAG – 8).

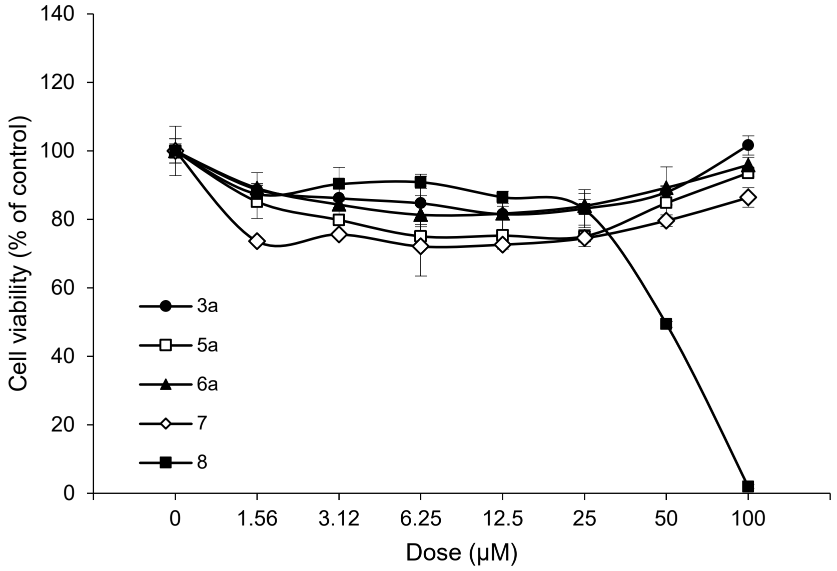

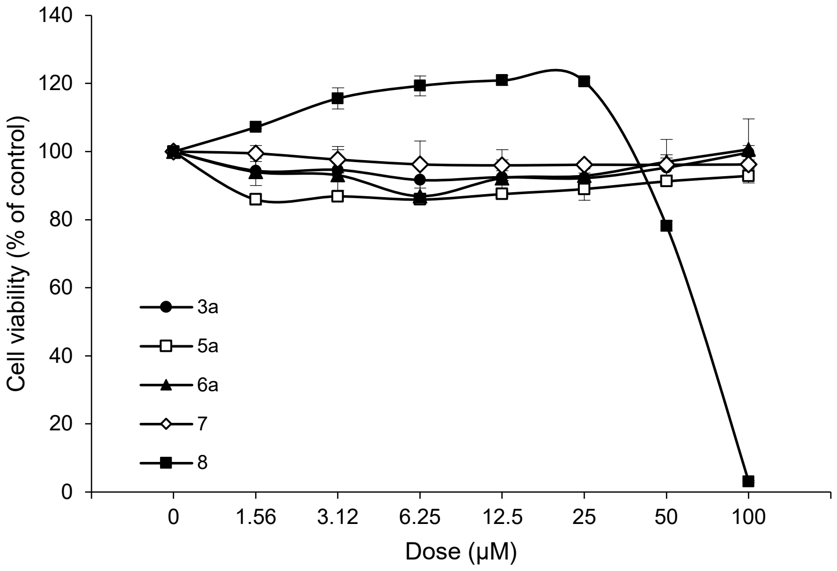

Figure S10 The dose-response curve for the activity towards A375 measured by MTT (upper) and CV (lower) tests (Zn-PLAG – 3a, Co-PLAG – 6a, Fe-PLAG – 5a, Cu-PLAG – 8).

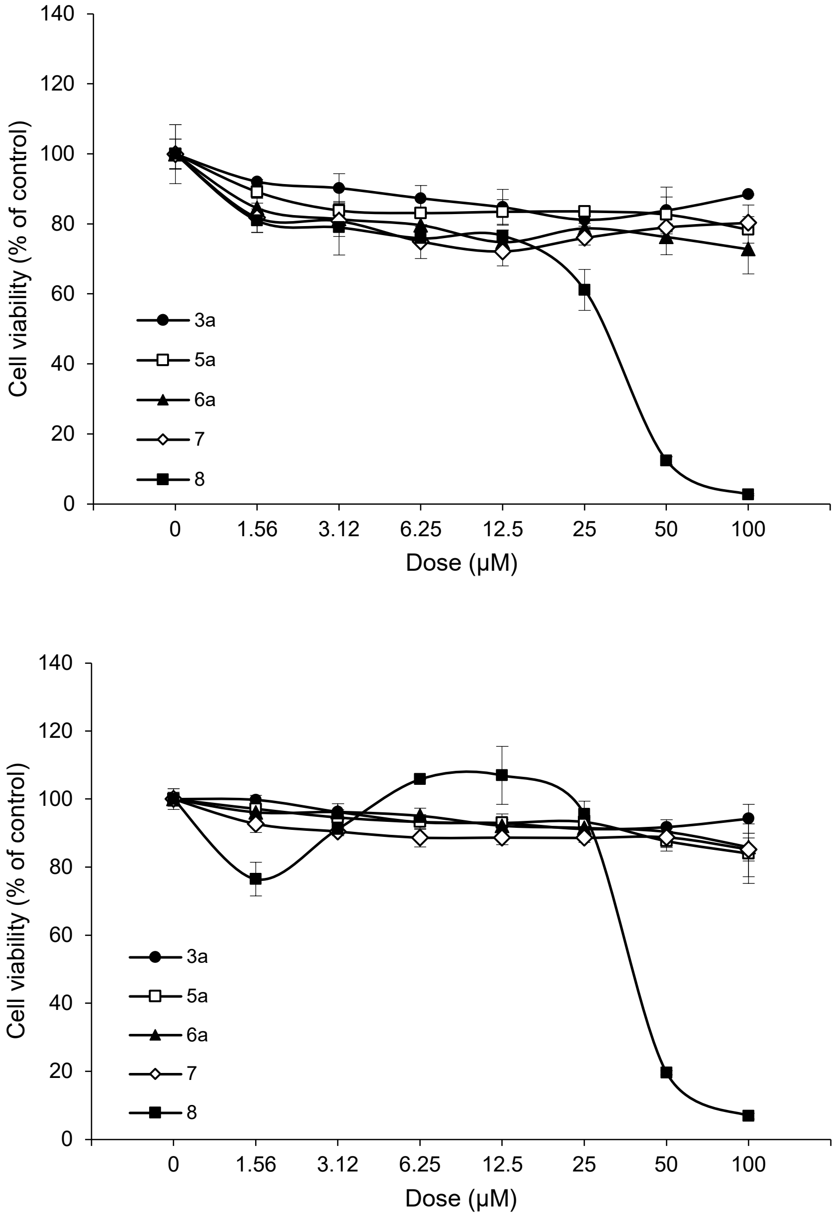

Figure S11 The dose-response curve for the activity towards A375 measured by MTT (upper) and CV (lower) tests (Zn-PLAG – 3a, Co-PLAG – 6a, Fe-PLAG – 5a, Cu-PLAG – 8).
